# Decoding the essence of two-character Chinese words: Unveiling valence, arousal, concreteness, familiarity, and imageability through word norming

**DOI:** 10.3758/s13428-024-02437-w

**Published:** 2024-05-15

**Authors:** Yuen-Lai Chan, Chi-Shing Tse

**Affiliations:** 1 Department of Educational Psychology, The Chinese University of Hong Kong, New Territories, Hong Kong, China; 2 Centre for Learning Sciences and Technologies, The Chinese University of Hong Kong, Hong Kong, China

**Keywords:** Chinese word, Megastudy, Norming, Valence, Visual word recognition

## Abstract

Investigation of affective and semantic dimensions of words is essential for studying word processing. In this study, we expanded Tse et al.’s (Behav Res Methods 49:1503–1519, 2017; Behav Res Methods 55:4382–4402, 2023) Chinese Lexicon Project by norming five word dimensions (valence, arousal, familiarity, concreteness, and imageability) for over 25,000 two-character Chinese words presented in traditional script. Through regression models that controlled for other variables, we examined the relationships among these dimensions. We included ambiguity, quantified by the standard deviation of the ratings of a given lexical variable across different raters, as separate variables (e.g., valence ambiguity) to explore their connections with other variables. The intensity–ambiguity relationships (i.e., between normed variables and their ambiguities, like valence with valence ambiguity) were also examined. In these analyses with a large pool of words and controlling for other lexical variables, we replicated the asymmetric U-shaped valence–arousal relationship, which was moderated by valence and arousal ambiguities. We also observed a curvilinear relationship between valence and familiarity and between valence and concreteness. Replicating Brainerd et al.’s (J Exp Psychol Gen 150:1476–1499, 2021; J Mem Lang 121:104286, 2021) quadratic intensity–ambiguity relationships, we found that the ambiguity of valence, arousal, concreteness, and imageability decreases as the value of these variables is extremely low or extremely high, although this was not generalized to familiarity. While concreteness and imageability were strongly correlated, they displayed different relationships with arousal, valence, familiarity, and valence ambiguity, suggesting their distinct conceptual nature. These findings further our understanding of the affective and semantic dimensions of two-character Chinese words. The normed values of all these variables can be accessed via https://osf.io/hwkv7.

Databases containing subjective ratings of lexico-semantic characteristics play a crucial role in psycholinguistic research. To establish standardized databases for a large pool of words, norming studies are commonly conducted (e.g., Altarriba et al., 1999; Balota et al., 2001; Juhasz & Yap, 2013; Schock et al., 2012; Sutton & Altarriba, 2016; Yao et al., 2017). Participants are instructed to rate individual words on various dimensions, such as concreteness and valence. Using these normed values, researchers select appropriate stimuli to control for and/or manipulate lexical variables in their experiments (e.g., Balota et al., 2007; Coltheart, 1981; Vigliocco et al., 2014; Warriner et al., 2013). The normed datasets are reusable across studies, saving time and effort for researchers while also facilitating comparisons of the findings across experiments (e.g., Keuleers & Balota, 2015). For instance, researchers examine the influence of lexical variables on lexical decision performance normed in megastudies to address research questions in visual word recognition (e.g., Kuperman et al., 2014; Su et al., 2023a, 2023b; Tse & Yap, 2018; Yap & Balota, 2009).

Bradley and Lang’s (1999) Affective Norms for English Words (ANEW) database normed 1034 words using the nine-point Self-Assessment Manikin (SAM) rating scale, measuring three emotional dimensions: valence (very pleasant to very unpleasant), arousal (very excited to very calm), and dominance (being in control to dominated). They revealed a symmetric U-shaped relationship between valence and arousal: words rated as positive or negative in valence generally had higher arousal ratings compared to those rated as neutral. Dominance was found to be highly correlated with valence (e.g., Imbir, 2016; Moors et al., 2013), such that researchers have often focused on valence and arousal, but not dominance, in their studies (e.g., Ćoso et al., 2019; Xu et al., 2022; Yao et al., 2017). The ANEW database has been widely used in studies involving emotion words and expanded and/or translated into other languages, including Chinese (e.g., Ho et al., 2015), Dutch (e.g., Moors et al., 2013), English (e.g., Warriner et al., 2013), Finnish (e.g., Söderholm et al., 2013), French (e.g., Monnier & Syssau, 2014), German (e.g., Võ et al., 2009), Indonesian (e.g., Sianipar et al., 2016), Italian (e.g., Montefinese et al., 2014), Portuguese (e.g., Soares et al., 2012), Polish (e.g., Imbir, 2016), and Spanish (e.g., Hinojosa et al., 2016). A summary of these studies is listed in the Appendix. The review below primarily focuses on the findings of Chinese norms, but we also incorporate results of non-Chinese norms when discussing our current findings.

In the current study, we focus on two-character Chinese words in traditional script. Two-character words, e.g., 朋友friend, constitute more than 70% of Chinese words (e.g., Institute of Language Teaching and Research, 1986). Traditional script refers to the original form of written characters that were used for centuries and is of popular use in Hong Kong, Taiwan, and Macau. Simplified script, on the other hand, was introduced in mainland China in 1960s for simplifying some characters by reducing their stroke counts and transforming the shape of their components (e.g., 藥 and 药 [medicine] in traditional and simplified script, respectively, Liu & Hsiao, 2012). The cultural and historical contexts associated with the two scripts might lead to variations in the perception, interpretation, and emotional connotations of words For example, 城市 [city] in mainland China carries a positive meaning, as it emphasizes the benefits of rapid urbanization, modern infrastructure, and economic development. In contrast, in Hong Kong, its word valence is more neutral due to complex challenges associated with urban density, fast-paced living, and the delicate balance between preserving heritage and embracing modernity in the city’s unique blend of Western and Chinese influences.

Previous Chinese norming studies with relatively large word pool (*N*s = 1,100–11,310), which were conducted in mainland China, presented words in simplified script (Lv et al., 2023; Wang et al., 2008; Xu et al., 2022; Yao et al., 2017). However, those involving traditional script, which were conducted in Hong Kong, used rather small word pools (*N* < 300, Ho et al., 2015; Yee, 2017). In the current study, we aimed to establish a much larger norm for Chinese words presented in traditional script, using young adults in Hong Kong as participants, the same population as in Tse et al.’s (2017, 2023) Chinese Lexicon Project. This will allow future researchers to examine the valence effect on visual word recognition of two-character Chinese words, based on Tse et al.’s normed lexical decision and naming data.

## Affective norms of Chinese words

Various studies have normed emotion variables of Chinese words and explored their relationships with other lexico-semantic variables (Ho et al., 2015; Lv et al., 2023; Wang et al., 2008; Xu et al., 2022; Yao et al., 2017; Yee, 2017, see Appendix). Most studies involved only two-character words, while Xu et al. included two-, three-, and four-character words and Lv et al. encompassed a wider spectrum, ranging from single characters to multiple-character words and phrases. While Xu et al. normed the emotion variables for 11,310 words (with the majority in two-character words, *N = *9,774), the number of words involved in other studies was much lower (*N*s = 160, 4,030^1^, 1,500, 1,100, and 292, for Ho et al., Lv et al., Wang et al., Yao et al., and Yee, respectively). Some studies focused on specific word types (nouns in Wang et al.; nouns, adjectives, and verbs in Yao et al.; low-/medium-frequency nouns in Yee; high-frequency words and phrases in Lv et al.; Ho et al. and Xu et al. did not specify the word type). While Ho et al. and Xu et al. collected the ratings from adolescents aged 12–17 and adults spanning a wide age range of 18–62, respectively, others recruited undergraduate students as their raters. Yee presented their words in traditional script, Ho et al. did that in both traditional and simplified scripts for separate groups of raters, and all other studies presented the words in simplified script. In the following, we summarize the findings of these norming studies (see Appendix for more details), although most of them (except Yao et al. and Yee) did not examine all relationships between emotion and lexico-semantic variables.

### Relationships among emotion and lexico-semantic variables

#### **Valence–arousal**

The valence–arousal relationship was shown to be symmetric U-shaped in Ho et al. (2015), Wang et al. (2008), Xu et al. (2022), and Yee (2017), that is, words with more extreme valence being more arousing than those with less extreme valence. However, when Yao et al. (2017) analyzed this by categorizing words into negative (1–4), neutral (4–6), or positive (6–9) in valence ratings, they found an asymmetric valence–arousal relationship: the increase in arousal was sharper for negative words than positive words. Negative words tend to elicit stronger arousal due to their association with potential danger, whereas positive stimuli may often be associated with feelings of safety. However, while Lv et al. (2023) also found an asymmetric valence–arousal relationship, it was different from Yao et al.’s one: positive words were more arousing than negative words.

#### **Valence–familiarity**

Familiarity reflects an individual’s prior exposure or experience with a word and is often quantified by participants’ ratings on how familiar they feel towards a specific word. The relationship between valence and familiarity was positive in Wang et al. (2008) and Yee (2017). Consistent with the mere exposure effect (e.g., Zajonc, 2001), more familiar words tend to evoke more positive evaluation. However, this explanation was at odds with Yao et al.’s (2017) findings that both highly positive *and* negative words were rated more familiar than the weakly positive and negative words, as shown by a quadratic valence–familiarity relationship after the squared valence term was included in the regression model.

#### **Valence–concreteness**

Concreteness refers to the degree to which a word can be associated with specific sensory experience or mental images. Yee (2017) reported a significant yet weakly negative linear relationship between valence and concreteness (*r* = −.12), with positive words being slightly more abstract than neutral and negative words, whereas Xu et al. (2022) did not find this relationship (*r* = −.01, after adjusting the direction of correlation, as Xu et al.’s concreteness scale was in reverse order to other studies). Yao et al. (2017) showed an inverted U-shaped, quadratic relationship between valence and concreteness, suggesting that emotion words, regardless of whether positive or negative, tend to be more abstract than neutral words (see also Lv et al., 2023). This is consistent with the embodiment view of the role of emotion in abstract words (e.g., Guasch et al., 2016; Kousta et al., 2011; Vigliocco et al., 2009; Vigliocco et al., 2014). Concrete and abstract words are semantically represented by experiential information (e.g., sensorimotor and affective experience) and linguistic information. The distinction between concrete and abstract words arises from the varying prevalence of experiential information. Concrete words place greater emphasis on sensorimotor information, whereas abstract words are more strongly associated with affective and linguistic knowledge. Thus, emotion words tend to be more abstract than neutral words.

#### **Valence–imageability**

Imageability refers to the ease with which a word can evoke mental image or sensory experience. Yee (2017) did not obtain any linear valence–imageability relationship (*r* = −.01). In contrast, after including the squared valence term in the regression model, Yao et al. (2017) showed an inverted U-shaped, quadratic valence–imageability relationship, showing that mental images could be formed more easily for neutral words than for positive and negative words.

#### **Arousal–familiarity**

Compared with word valence, the findings of word arousal were not as robust. Only Yee (2017) examined the arousal–familiarity relationship and obtained a nonsignificant correlation between them (*r* = −.11).

#### **Arousal–concreteness**

Yao et al. (2017) found a negative linear arousal–concreteness relationship, with highly arousing words being more abstract than non-arousing words, in line with Vigliocco et al.’s (2014) view that abstract words are more associated with affective information than concrete words. While this was replicated in Xu et al. (2022) (*r* = −.20, after adjusting the direction of correlation, as Xu et al.’s concreteness scale was in reverse order to other studies) and Lv et al. (2023), Yee (2017) did not find such a relationship (*r* = −.02).

#### **Arousal–imageability**

Yao et al. (2017) reported a weakly negative linear relationship between arousal and imageability (*r* = −.06). In contrast, Yee (2017) did not find any arousal–imageability relationship (*r* = .02).

Why did previous studies show mixed evidence for the relationships among emotion and lexico-semantic variables? First, while Yao et al. (2017) controlled for other lexical variables (e.g., concreteness and familiarity) in their analyses, all other studies reported either Pearson correlation or simple regression models for pairwise comparisons between emotion and lexico-semantic variables, without any controlling variables. In fact, even Yao et al. did not control any variables when examining the arousal–concreteness relationship. Given the correlations between emotion and lexico-semantic variables, it is important to test the relationship between target lexical variables after keeping others constant.

Second, the number of words and the word type involved in the norming studies are highly varied. While Xu et al. (2022) based their findings on more than 10,000 Chinese words, the word pools in other studies were all less than 4030. In some studies, the words were restricted to certain word types (e.g., low-/medium-frequency nouns in Yee, 2017). A larger pool of words with various word types and potentially more diverse range of values in emotion and lexico-semantic variables should be used to reveal a larger pattern of results.

Finally, the scales of emotion and lexico-semantic variables were not the same across studies. Yao et al. (2017) used the typical nine-point SAM scale with pictorial figures for valence and arousal (e.g., Bradley & Lang, 1999). While Yee also used the SAM scale, she converted the nine-point scale to the five-point scale. Xu et al. (2022) used a seven-point scale for valence, ranging from extremely negative (−3) to neutral (0) to extremely positive (+3), and a five-point scale for arousal, ranging from very low arousal (0) to very high arousal (4). Lv et al. (2023) used seven-point scales, ranging from extremely negative (1) to neutral (4) to extremely positive (7), and categorized valence as negative (1–3), neutral (3–5), and positive (5–7) in their analyses. These differences in the bipolarity and range of rating scales might contribute to the discrepancies in the relationships among lexical variables. In the current study, we used the typical nine-point SAM scales with pictorial figures for valence and arousal ratings, with a wide range of values, to reveal a full picture of the relationships among lexical variables.

Other than the relationship between emotion variables and lexico-semantic variables, we consider the interrelationships among lexico-semantic variables. The correlations among concreteness, imageability, and familiarity were positive in Yao et al. (2017) and Yee (2017). According to Paivio’s (1991) dual-coding theory, information can be encoded as verbal, linguistic representation and nonverbal, imaginal representation. The strong concreteness–imageability relationship (.78 in Yao et al.; .88 in Yee) showed that concrete words are encoded and retrieved using both verbal and imagery codes, while abstract words rely more on verbal codes and are more difficult to visualize in mental images. The moderate positive correlations between concreteness and familiarity (.54 in Yao et al.; .34 in Yee) and between imageability and familiarity (.34 in Yao et al.; .41 in Yee) suggest that concrete and highly imageable words tend to be more familiar than abstract and difficult-to-image words.

## Valence ambiguity

Apart from valence, arousal, concreteness, imageability, and familiarity, we examined a novel lexical variable, which to our knowledge has never been investigated in two-character Chinese words. The valence of a word can be ambiguous due to personal experience. For example, “dog” could be perceived as positive for some individuals but negative for others who have been bitten by a dog. Previous works have often overlooked this uncertainty in self-reported valence. Brainerd (2018, see also Brainerd et al., 2021a, 2021b; Mattek et al., 2017) has quantified the standard deviation of valence ratings across different raters and labeled that as the valence ambiguity of a word. He found that words with higher valence ambiguity exhibited a weaker valence–arousal relationship for both negative and positive words, which was proposed as the *emotional-ambiguity hypothesis* (Brainerd, 2018).

Brainerd and associates (2021a, 2021b) utilized two word norms (Bradley & Lang, 1999; Warriner et al., 2013) to test the emotional-ambiguity hypothesis. They found that the correlation between arousal and valence was the strongest when valence ambiguity was the lowest and the correlation decreased linearly when valence ambiguity increased. Such a relationship was stronger in negative words than in positive words. Brainerd et al. (2021b) also found that the standard deviation of arousal (i.e., arousal ambiguity) could moderate the valence–arousal relationship. Brainerd et al. (2021a) further showed that valence ambiguity had a curvilinear relationship with valence rating, suggesting that valence ambiguity is a variable distinct from valence (see also Chang & Brainerd, 2023). By considering the mean rating as a type of intensity variable, Brainerd and his colleagues (2021a, 2021b) postulated a quadratic intensity–ambiguity relationship, which may occur in valence, arousal, and lexico-semantic variables, such as concreteness, familiarity, and imageability. They proposed a categorical/quantitative model to explain this intensity–ambiguity relationship. Participants tend to make categorical judgments when rating words with extreme values (i.e., highest intensity), but fine-grained quantitative judgments when rating words with values at the mid-range, resulting in a quadratic relationship between intensity and ambiguity. In the current study, we investigated whether the intensity–ambiguity relationships would occur in a large pool of two-character Chinese words in Tse et al. (2017, 2023).

## The present study

We conducted a norming study and developed a database of emotion (valence and arousal) and lexico-semantic variables (concreteness, familiarity, and imageability) for 25,000+ two-character Chinese words in traditional script. This large word pool was adopted from Tse et al. (2017, 2023), which normed the behavioral measures (reaction time and accuracy) of participants’ lexical decision and speeded naming responses for all these words. For the emotion variables, we employed Bradley and Lang’s (1999) nine-point SAM scale to enlarge the range of values to capture the subtle effects (e.g., curvilinear relationship between valence and arousal) and make it easier to compare our findings with those obtained in other languages, e.g., English. We normed lexico-semantic variables to test the relationships among these variables and the emotion variables. This sheds light on the mixed evidence reported in previous studies that used much smaller word pools and more restricted sets of lexical variables (e.g., Yao et al., 2017; Yee, 2017). Apart from interrelationships among emotion and lexico-semantic variables, we took inspiration from Brainerd and his colleagues’ (2018, 2021a, 2021b) work on the valence ambiguity of English words and addressed further questions in our analyses of two-character Chinese words: How could the valence ambiguity relate to valence and other lexical variables? Can the valence–arousal relationship be moderated by valence ambiguity and arousal ambiguity (emotional-ambiguity hypothesis)? Can the quadratic intensity–ambiguity relationship be revealed in arousal and lexico-semantic variables? We computed the ambiguity (i.e., standard deviation of the ratings across raters) of valence and arousal variables to examine how valence ambiguity could be related to arousal and lexico-semantic variables and to test the emotional-ambiguity hypothesis, that is, the role of valence ambiguity and arousal ambiguity in the valence–arousal relationship. We also computed the ambiguity of lexico-semantic variables and tested whether Brainerd et al.’s (2021a, 2021b) quadratic intensity–ambiguity relationship on arousal, familiarity, concreteness, and imageability could be generalized to two-character Chinese words.

To recapitulate, there are five goals of the current research. First, we normed the ratings of valence, arousal, familiarity, concreteness, and imageability of over 25,000 two-character Chinese words (Tse et al., 2017), presented in traditional script, in Hong Kong. Second, we examined the interrelationships among these variables and compared those with previous studies that were based on relatively fewer words (e.g., Yao et al., 2017; Yee, 2017), while other variables and their ambiguities were controlled. Third, we explored the role of valence ambiguity in the relationships among the various lexico-semantic variables. Fourth, we examined the emotional-ambiguity hypothesis that valence and arousal ambiguities could influence the valence–arousal relationship. Fifth, we tested the intensity–ambiguity relationship for emotion and lexico-semantic variables to replicate the findings from Brainerd et al.’s study (2021a, 2021b). These findings could advance our understanding of the affective and semantic dimensions of two-character Chinese words.

## Method

### Participants

A total of 1,080 native Cantonese-speaking students from the Chinese University of Hong Kong, the same population as in Tse et al. (2017), were recruited and randomly divided into three groups, who were given valence, arousal, and lexico-semantic (concreteness, familiarity, and imageability) online rating tasks, respectively. Participants who reported system errors (*N* = 37), were left-handed (*N* = 2), or did not complete the tasks (*N* = 59) were replaced. Participants aged 17 and 34 years old (*N* = 2) were replaced to ensure that our age range (18–25) was comparable to Tse et al.’s. For the valence, arousal, and lexico-semantic groups, 64.7% (*N* = 233), 67.2% (*N* = 242), and 64.4% (*N* = 232) of participants were female, and the mean age was 19.95, (*SD* = 1.52), 19.82 (*SD* = 1.46), and 19.66 (*SD* = 1.38), respectively. Those of the valence and arousal groups received HKD 60 (~USD 7.50) as monetary compensation for their participation. For the lexico-semantic group, the blocks of concreteness, imageability, and familiarity rating tasks were counterbalanced in order and the URLs of the tasks were sent to participants one by one upon completion. These participants received HKD 300 (~USD 37.50) as monetary compensation.

### Materials and procedure

The 25,281 words from Tse et al.’s (2017) Chinese Lexicon Project were divided into 18 lists of 1405–1406 words each. Each list was assigned to 20 participants in each of the valence, arousal, and lexico-semantic groups. Due to the COVID-19 outbreak, all ratings were collected online using PsychoPy (Peirce et al., 2019) via Pavlovia.org. Participants received a URL for their rating task via email. They signed the informed consent form at the beginning of the task.

For valence and arousal ratings, we adapted Bradley and Lang’s (1999) instruction and nine-point SAM rating scale (1 = extremely negative/calm; 9 = extremely positive/excited). For the ratings of lexico-semantic variables, the instructions were based on Yee (2017) with a wider, seven-point Likert scale, with 1 indicating very abstract, very unfamiliar, and difficult to form a mental image, respectively, and 7 indicating very concrete, very familiar, and easy to form a mental image, respectively. The words were presented one at a time and stayed on the screen until participants responded by pressing a numeric key. They were told to rate the words as quickly as possible based on their first impression. They were given examples and detailed definitions of the variables in the rating tasks. We did not provide an “I don’t know” option because it is possible that participants might respond “I don’t know” when they actually knew the word but were just uncertain how to rate that on a specific dimension. The extent to which a word was familiar to our target population could be reflected by our normed familiarity ratings, as well as the lexical decision and naming accuracy normed in Tse et al. (2017, 2023).

## Results

The mean ratings and standard deviations (*SD*s) of all lexical variables normed for 25,281 words are available at: https://osf.io/hwkv7. Each word was rated by 20 participants. All statistical analyses were performed by R in RStudio (2022.07.1, Build 554). Table 1 shows descriptive statistics of valence, arousal, concreteness, familiarity, and imageability ratings, as well as their ambiguity measures (i.e., the standard deviation of the ratings of a given lexical variable across different raters).

**Table 1:** Descriptive statistics (*N* = 25,281)

Ratings	Mean	*SD*	Range	Skewness	Kurtosis
Valence (9-point scale)	5.02	0.97	1.40-7.95	-0.42	2.92
Arousal (9-point scale)	3.81	0.89	1.60-7.60	0.66	3.20
Familiarity (7-point scale)	5.77	0.49	2.75-6.90	-0.97	4.61
Concreteness (7-point scale)	4.60	0.72	2.20-6.65	-0.004	2.42
Imageability (7-point scale)	4.21	0.87	1.85-6.65	0.17	2.25
Valence ambiguity	1.30	0.30	0.22-2.63	0.22	3.11
Arousal ambiguity	2.18	0.33	0.86-3.33	-0.21	2.95
Familiarity ambiguity	1.31	0.32	0.31-2.62	0.33	2.96
Concreteness ambiguity	1.51	0.26	0.51-2.46	0.04	2.72
Imageability ambiguity	1.63	0.25	0.59-2.46	-0.17	2.97

**Fig. 1:**
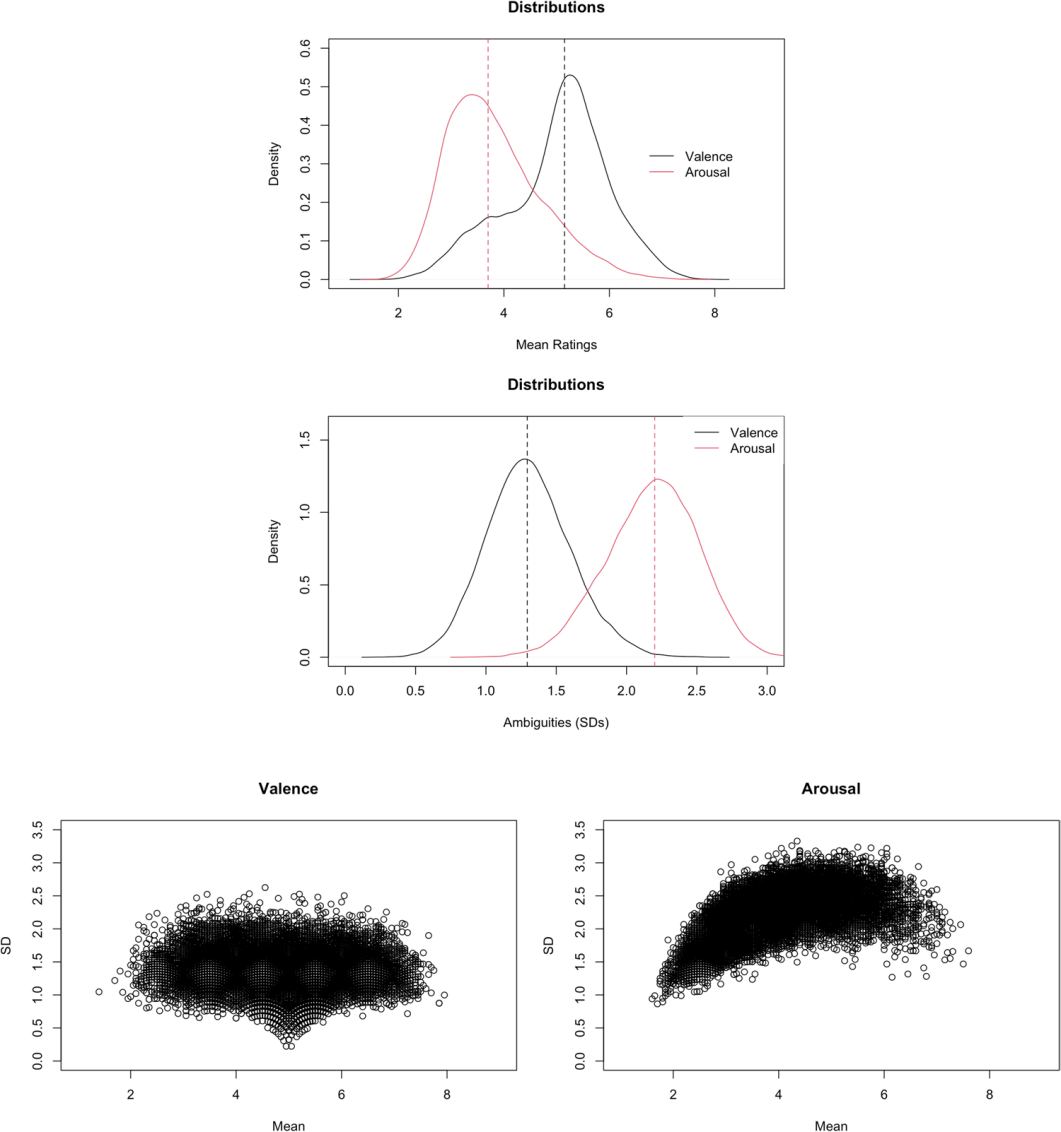
Top: Distributions of valence and arousal ratings. Middle: Distributions of valence and arousal ambiguities. Bottom: Scatterplots for the variability in valence (left) and arousal (right) ratings. Dotted lines indicate the medians

**Fig. 2:**
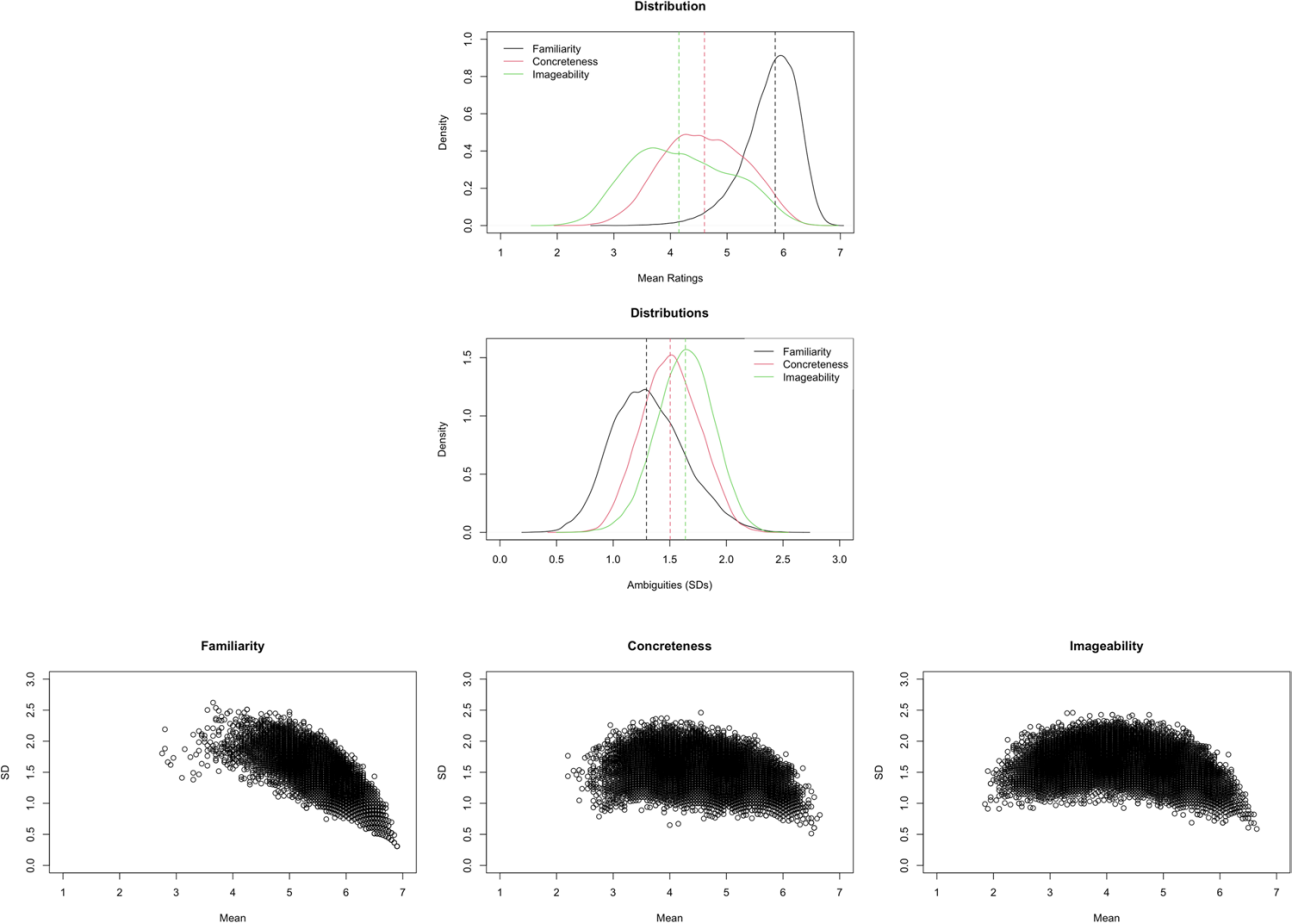
Top: Distributions of familiarity, concreteness, and imageability ratings. Dotted lines indicate the medians. Middle: Distributions of familiarity, concreteness, and imageability ambiguities. Bottom: Scatter plots for the variability in concreteness (left), familiarity (middle), and imageability (right)

Figures 1 and 2 show the plots of distributions and rating variability (*SD*) of normed variables. The valence ratings were about normally distributed, with 56.83% of words rated above the mean. The ratings were least variable for words in the middle range. The distribution of arousal ratings was slightly positively skewed, with 43.45% of words rated above the mean. The ratings were least variable for words that were least arousing or very calm. The valence ambiguity (i.e., *SD* of valence ratings across 20 raters), was about normally distributed, with 48.55% of words having valence ambiguity scores above the mean. The arousal ambiguity (i.e., *SD* of arousal ratings across 20 raters) was about normally distributed, with 52.07% of words having arousal ambiguity scores above the mean. The familiarity rating was negatively skewed, with 55.54% of words rated above the mean. The variability decreased as familiarity increased, reflecting that the majority of our words were highly familiar to our participants. The concreteness and imageability ratings were about normally distributed, with 50.20% and 46.97% of words rated above the mean, respectively. The variability in these two ratings was similar, where words located at the two ends of the scale had lower variability, especially at the highest ends. The ambiguity of familiarity, concreteness, and imageability (i.e., *SD*s of familiarity, concreteness, and imageability ratings across 20 raters) was about normally distributed, with 47.58%, 49.08%, and 51.90% of words having familiarity ambiguity, concreteness ambiguity, and imageability ambiguity scores above the means, respectively.

### Reliability of the ratings

Following previous studies (e.g., Yao et al., 2017), interrater reliability of valence, arousal, concreteness, familiarity, and imageability ratings was calculated by split-half correlations and corrected with the Spearman–Brown formula. For each rating, 20 participants were divided into two equal groups based on odd/even participant numbers. The corrected correlation was higher for valence (.91) than for arousal (.74), consistent with previous studies (e.g., Eilola & Havelka, 2010; Warriner et al., 2013; Yao et al., 2017). The corrected correlations for concreteness, familiarity, and imageability were .80, .69, and .84 respectively. To test whether our ratings were comparable to previous norming studies in Chinese, we conducted correlation analyses on our data and other Chinese norms. Our valence and arousal ratings were moderately to strongly associated with those in Yee (2017, *N* = 283 in common) (+.90 and +.63) and Xu et al. (2022, *N* = 9,125 in common) (+.87 and +.62). This was the case even though our scales (nine-point SAM scale) differed from theirs (bipolar valence scale in Xu et al. and five-point SAM scale in Yee). Our concreteness, familiarity, and imageability ratings were moderately associated with those in Yee (2017) (+.53, +.48, and +.48). Our concreteness rating was also strongly associated with the one in Xu and Li (2020, *N* = 8,675 in common, −.78, the opposite sign as their scale was in a reverse direction to ours), while our imageability was strongly associated with the one in Su et al. (2023b, *N* = 9,125 in common, +.77). (These latter two studies did not norm any emotion variables.) In contrast, our ratings were weakly associated with Yao et al. (2017, *N* = 1100 in common) (+.38, +.21, +.01, +.10, and +.02 for valence, arousal, concreteness, familiarity, and imageability, respectively). Our familiarity rating was also weakly associated with the one in Su et al. (2023a, *N* = 15,228 in common, +.07). These could be attributed to two factors. First, there are some differences in the instructions among studies. For example, in concreteness ratings, Yao et al. asked their participants to think whether words could be associated with mental images in some scenarios, whereas we told participants to rate based on definitions and examples (see also, e.g., Xu & Li, 2020). Second, Yao et al.’s and Su et al.’s raters were recruited from mainland China, whereas we recruited our raters in Hong Kong, that is, the same as those in Yee. There could be a difference between the raters from mainland China and Hong Kong in their familiarity for Chinese words, highlighting the importance of developing separate word norms for two populations. However, these explanations could not explain why our valence and arousal ratings were strongly associated with Xu et al., in which the ratings were also collected using participants in mainland China.

### Relationships among lexical variables

Multiple regression analyses were conducted, with arousal, familiarity, concreteness, imageability, valence ambiguity, arousal ambiguity, familiarity ambiguity, concreteness ambiguity, and imageability ambiguity used as the outcome variables in separate models (see Tables 2, 3 and 4). To control for the potential confound of frequency effect (e.g., Brainerd & Bookbinder, 2019; Citron et al., 2014), we included log-transformed character and word frequency based on subtitle contextual diversity (Cai & Brysbaert, 2010), which was shown to better predict word recognition performance than other frequency measures (e.g., Tse et al., 2017). Multiple regression analyses were run on 20,218 (80.0%) words with available values of all lexico-semantic variables. All predictor variables were centered and *z*-transformed to avoid potential multicollinearity problem. All variance inflation factors were low (< 3). Unlike most of the previous studies in Chinese (e.g., Yee, 2017), we controlled for the influence of other lexico-semantic variables and their ambiguity variables when analyzing our data. The adjusted *R*^2^ is quoted in the analyses reported below. We discuss our findings with those reported in Chinese norms, as well as the patterns reported in other languages, such as English and Spanish (see the summary table in Appendix Table 5 for more details).

**Table 2:** Standardized regression coefficients β and standard error in parentheses of Models 1–10 (*N* = 20,218)

Predictor variables	Outcome variable									
	Arousal		Familiarity		Concreteness		Imageability		Valence ambiguity	
	Model 1	Model 2	Model 3	Model 4	Model 5	Model 6	Model 7	Model 8	Model 9	Model 10
Log C1 frequency	−.043*** (.005)	−.019*** (.005)	.047*** (.004)	.051*** (.004)	.004 (.005)	.0005 (.005)	−.097*** (.005)	−.096*** (.005)	−.039*** (.007)	−.037*** (.007)
Log C2 frequency	−.040*** (.005)	−.018*** (.005)	.038*** (.004)	.042*** (.004)	−.013* (.005)	−.016** (.005)	−.104*** (.005)	−.103*** (.005)	−.049*** (.007)	−.047*** (.007)
Log word frequency	.061*** (.005)	.047*** (.005)	.149*** (.005)	.146*** (.005)	.023*** (.006)	.024*** (.006)	−.047*** (.006)	−.047*** (.006)	−.014 (.008)	−.015 (.008)
Valence	−.374*** (.005)	−.299*** (.005)	−.014** (.005)	−.007 (.005)	−.116*** (.006)	−.120*** (.006)	.050*** (.006)	.052*** (.006)	.070*** (.008)	.073*** (.008)
Valence^2^	-	.195*** (.004)	-	.050*** (.004)	-	−.035*** (.004)	-	.010* (.004)	-	.020*** (.006)
Arousal	-	-	−.049*** (.006)	−.080*** (.007)	−.223*** (.007)	−.200*** (.008)	.042*** (.007)	.036*** (.008)	.330*** (.010)	.317*** (.010)
Familiarity	−.064*** (.008)	−.093*** (.008)	-	-	−.063*** (.008)	−.056*** (.008)	.175*** (.008)	.173*** (.008)	.068*** (.011)	.064*** (.011)
Concreteness	−.203*** (.007)	−.160*** (.006)	−.044*** (.006)	−.039*** (.006)	-	-	.563*** (.006)	.564*** (.006)	−.069*** (.009)	−.067*** (.009)
Imageability	.041*** (.007)	.031*** (.006)	.131*** (.006)	.128*** (.006)	.603*** (.006)	.602*** (.006)	-	-	.051*** (.010)	.051*** (.010)
Valence ambiguity	.167*** (.005)	.140*** (.005)	.026*** (.004)	.025*** (.004)	−.038*** (.005)	−.037*** (.005)	.027*** (.005)	.026*** (.005)	-	-
Arousal ambiguity	.462*** (.005)	.413*** (.005)	−.006 (.005)	−.003 (.005)	.052*** (.006)	.050*** (.006)	.016** (.006)	.017** (.006)	.020* (.008)	.021* (.008)
Familiarity ambiguity	−.099*** (.008)	−.093*** (.007)	−.707*** (.005)	−.702*** (.005)	−.033*** (.008)	−.032*** (.008)	−.011 (.008)	−.012 (.008)	.048*** (.011)	.047*** (.011)
Concreteness ambiguity	−.028*** (.005)	−.042*** (.005)	−.006 (.005)	−.010* (.005)	−.147*** (.005)	−.143*** (.005)	−.133*** (.005)	−.134*** (.005)	.026*** (.007)	.024*** (.007)
Imageability ambiguity	.031*** (.005)	−.029*** (.005)	.068*** (.004)	.067*** (.004)	−.025*** (.005)	−.025*** (.005)	−.147*** (.005)	−.147*** (.005)	.022** (.007)	.022** (.007)

***p < .001, **p < .01, *p < .05; valence^2^ = squared term of valence; C1= first character, C2 = second character

**Table 3:** Standardized regression coefficients β and standard errors (in parentheses) of Model 2a and 2b (full scale: *N* = 20,218; negative subfile: *N* = 7,390; positive subfile: *N* = 12,288)

Predictor variables	Outcome variable					
	Arousal - Model 2a			Arousal - Model 2b		
	Full scale	Negative subfile	Positive subfile	Full scale	Negative subfile	Positive subfile
Log C1 frequency	−.019*** (.005)	−.013 (.008)	−.025*** (.007)	−.016*** (.005)	−.009 (.007)	−.025*** (.007)
Log C2 frequency	−.017*** (.005)	−.002 (.008)	−.030*** (.007)	−.014** (.005)	.002 (.007)	−.031*** (.007)
Log word frequency	.046*** (.005)	.069*** (.009)	.039*** (.007)	.047*** (.005)	.067*** (.009)	.039*** (.007)
Valence	−.289*** (.005)	−.505*** (.009)	.036*** (.009)	−.341*** (.005)	−.509*** (.009)	.035*** (.009)
Valence^2^	.196*** (.004)	.028*** (.008)	.034*** (.005)	.240*** (.004)	.088*** (.008)	.035*** (.006)
Familiarity	−.091*** (.008)	−.135*** (.012)	−.060*** (.011)	−.090*** (.007)	−.133*** (.012)	−.061*** (.011)
Concreteness	−.159*** (.006)	−.155*** (.010)	−.185*** (.009)	−.149*** (.006)	−.143*** (.010)	−.186*** (.009)
Imageability	.030*** (.006)	.110*** (.011)	−.030** (.010)	.031*** (.006)	.099*** (.010)	−.029** (.010)
Valence ambiguity	.145*** (.006)	.166*** (.011)	.116*** (.009)	.136*** (.004)	.170*** (.008)	.117*** (.007)
Arousal ambiguity	.413*** (.005)	.262*** (.008)	.606*** (.007)	.475*** (.006)	.274*** (.011)	.610*** (.008)
Familiarity ambiguity	−.092*** (.007)	−.119*** (.012)	−.072*** (.011)	−.087*** (.007)	−.114*** (.011)	−.072*** (.011)
Concreteness ambiguity	−.042*** (.005)	−.047*** (.008)	−.049*** (.007)	−.044*** (.005)	−.054*** (.008)	−.050*** (.007)
Imageability ambiguity	.029*** (.005)	−.037*** (.008)	−.032*** (.007)	−.028*** (.005)	−.036*** (.007)	−.032*** (.007)
Valence ambiguity × valence	−.040*** (.005)	.001 (.008)	−.022** (.008)	-	-	-
Valence ambiguity × valence^2^	−.009* (.004)	.005 (.008)	−.001 (.006)	-	-	-
Arousal ambiguity × valence	-	-	-	.079*** (.005)	.173*** (.008)	−.008 (.008)
Arousal ambiguity × valence^2^	-	-	-	−.079*** (.003)	−.030*** (.007)	−.004 (.005)

***p < .001, **p < .01, *p < .05; valence^2^ = the quadratic (squared) term of valence; C1= first character, C2: second character

**Table 4:**
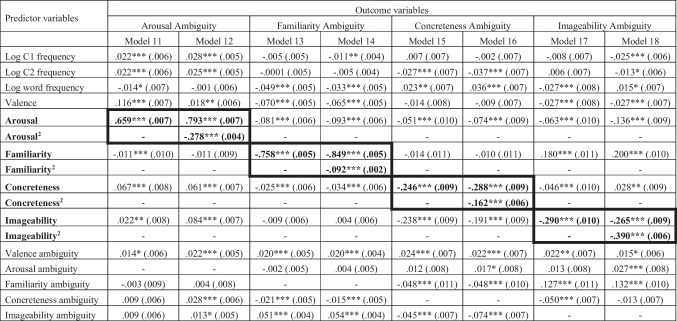
Standardized regression coefficients β and standard errors (in parentheses) of Models 11–18 (( N  = 20,218)

***p < .001, **p < .01, *p < .05; arousal^2^ = the quadratic (squared) term of arousal; familiarity^2^ = the quadratic (squared) term of familiarity; concreteness^2^ = the quadratic (squared) term of concreteness; imageability^2^ = the quadratic (squared) term of imageability; C1= first character, C2: second character. Bolded text and values represent the target variables and their coefficients

### Interrelationships among all normed variables and valence ambiguity

Table 2 presents the model summaries for the regression analyses with arousal, familiarity, concreteness, imageability, and valence ambiguity as outcome variables.

#### Valence–arousal

**Fig. 3:**
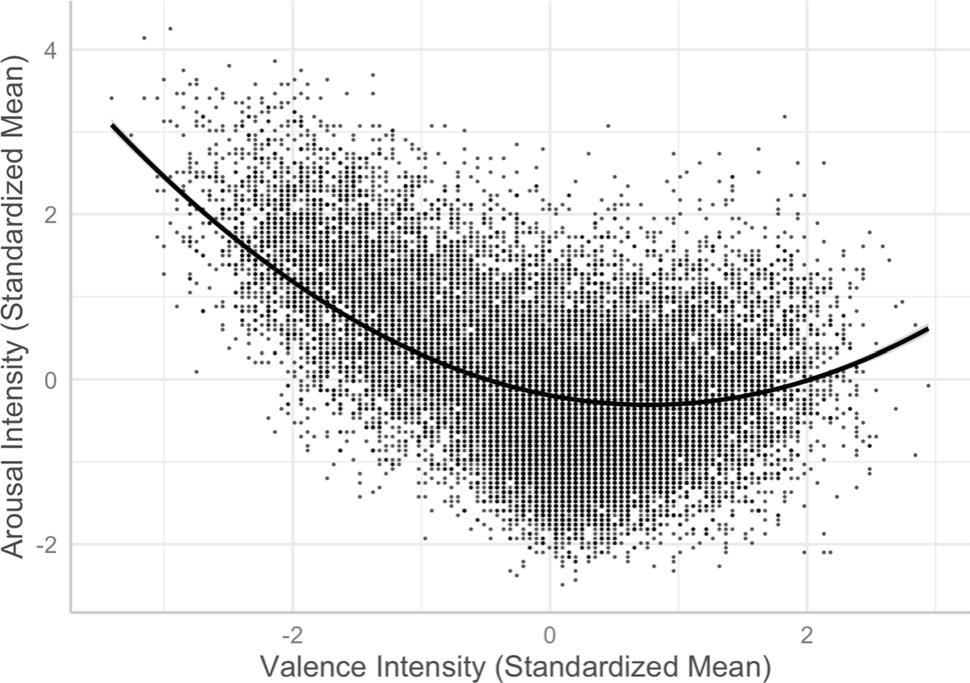
Valence–arousal relationship (Model 2)

The valence–arousal relationship was asymmetric U-shaped in that extremely negative words were rated more arousing than extremely positive words (see Fig. 3—only the model that accounted for more variance is depicted).^2^ This was consistent with Yao et al. (2017, see also, e.g., Citron et al., 2014, for English; Võ et al., 2009, for German; Imbir, 2016, for Polish; Guasch et al., 2016, for Spanish), but not Xu et al. (2022, see also, e.g., Warriner et al., 2013, for English; Eilola & Havelka, 2010, for Finnish). Relative to the linear model [Model 1, *R*^2^ = .5536, *F*(12,20205) = 2090, *p* < .001], adding the squared valence term significantly improved the model and accounted for more variance in arousal [Model 2, *R*^2^ = .6097, *F*(13,20204) = 2431, *p* < .001; *ΔR*^2^ = .056, *ΔF* = 2907.1, *p* < .001].

#### **Valence–familiarity**

**Fig. 4:**
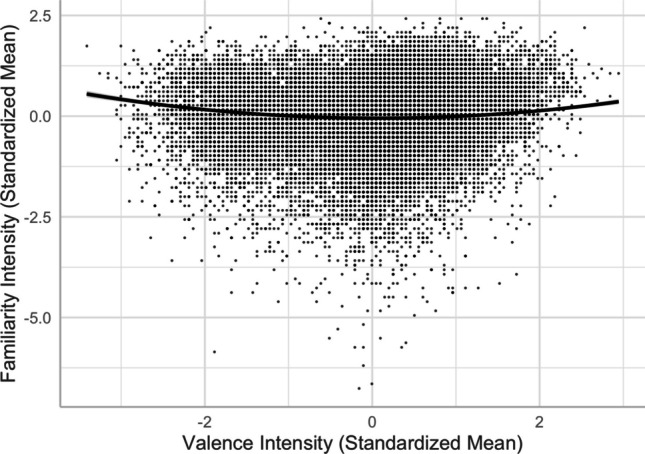
Valence–familiarity relationship (Model 4)

The linear valence–familiarity relationship, as depicted in Model 3 [*R*^2^ = .6588, *F*(12,20205) = 3254, *p* < .001], showed that more positive words were rated less familiar, which was inconsistent with Yee (2017), where more positive words were rated more familiar (see, e.g., Citron et al., 2014; Warriner et al., 2013, for similar findings in English). When ambiguity variables were not controlled, as done in Yee, we found a positive relationship, consistent with previous findings. Adding a squared valence term significantly improved the model and accounted for more variance in familiarity [Model 4, *R*^2^ = .662, *F*(13,20204) = 3047, *p* < .001; *ΔR*^2^ = .0032, *ΔF* = 191.93, *p* < .001], in line with Yao et al. (2017). As shown in Fig. 4, negative and positive words were more familiar than neutral words.

#### **Valence–concreteness**

**Fig. 5:**
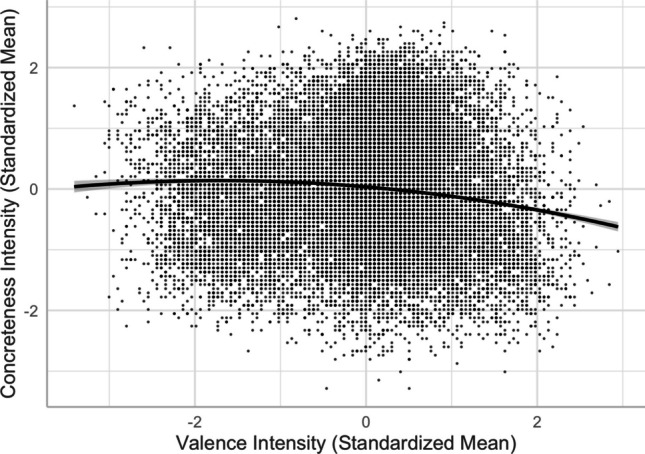
Valence–concreteness relationship (Model 6)

The linear negative valence–concreteness relationship, as depicted in Model 5 [*R*^2^ = .5094, *F*(12,20205) = 1750, *p* < .001], was consistent with Yee (2017) in that more positive words were rated less concrete (see also Hinojosa et al., 2016, for Spanish, but see Warriner et al., 2013, for English). Adding the squared valence term significantly improved the model and accounted for more variance in concreteness [Model 6, *R*^2^ = .5109, *F*(13,20204) = 1626, *p* < .001; *ΔR*^2^ = .0016, *ΔF* = 65.528, *p* < .001], partially consistent with Yao et al. (2017). As shown in Fig. 5, when word valence increased (became more positive), the words became more abstract. Unlike the symmetric inverted U-shaped relationship in Yao et al. (see also Sianipar et al., 2016, for Indonesian), negative words tended to be more concrete than positive words. This was not consistent with Vigliocco et al. (2014), who postulated that emotion words were more abstract than neutral words but did not distinguish the role of positive versus negative emotion in the semantic representation of abstract words.

#### **Valence–imageability**

**Fig. 6:**
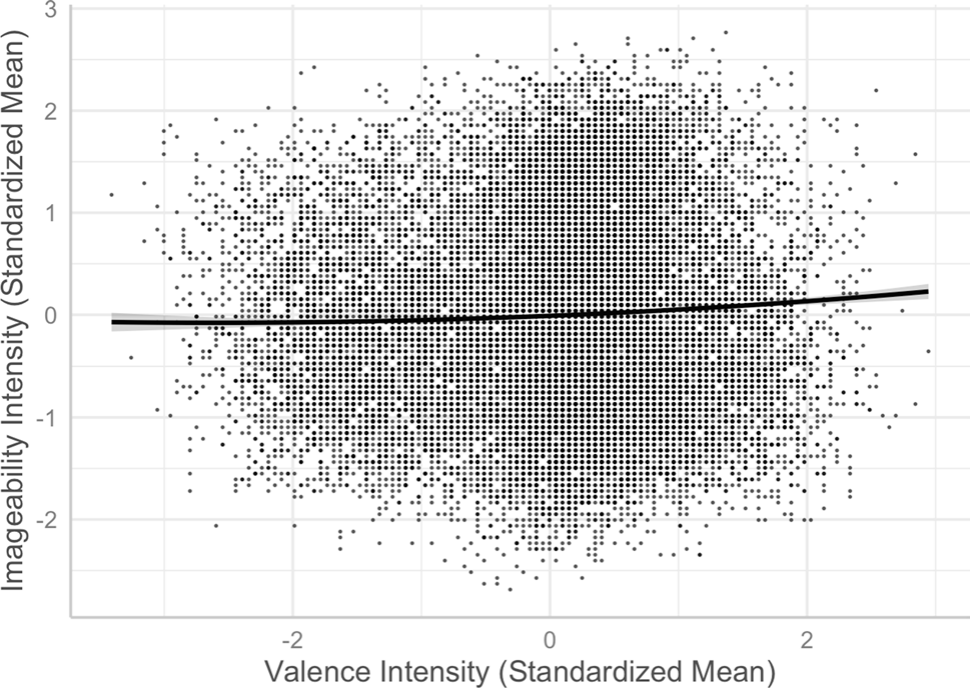
Valence–imageability relationship (Model 8)

In contrast to Yee (2017), we obtained a linear valence–imageability relationship [*R*^2^ = .5418, *F*(12,20205) = 1993, *p* < .001]. As depicted in Model 7, more positive words were rated more imageable (see also, e.g., Citron et al., 2014; Warriner et al., 2013, in English; and Imbir, 2016, Riegel et al., 2015, in Polish). Adding a squared valence term improved the model significantly [Model 8, *R*^2^ = .5419, *F*(13,20204) = 1840, *p* < .001; *ΔR*^2^ = .0002, *ΔF* = 5.5882, *p* < .05]. As shown in Fig. 6, positive words were more imageable than negative and neutral words, in contrast to Yao et al.’s pattern where both positive and negative words were less imageable than neutral words*.*

#### **Arousal–familiarity**

**Fig. 7:**
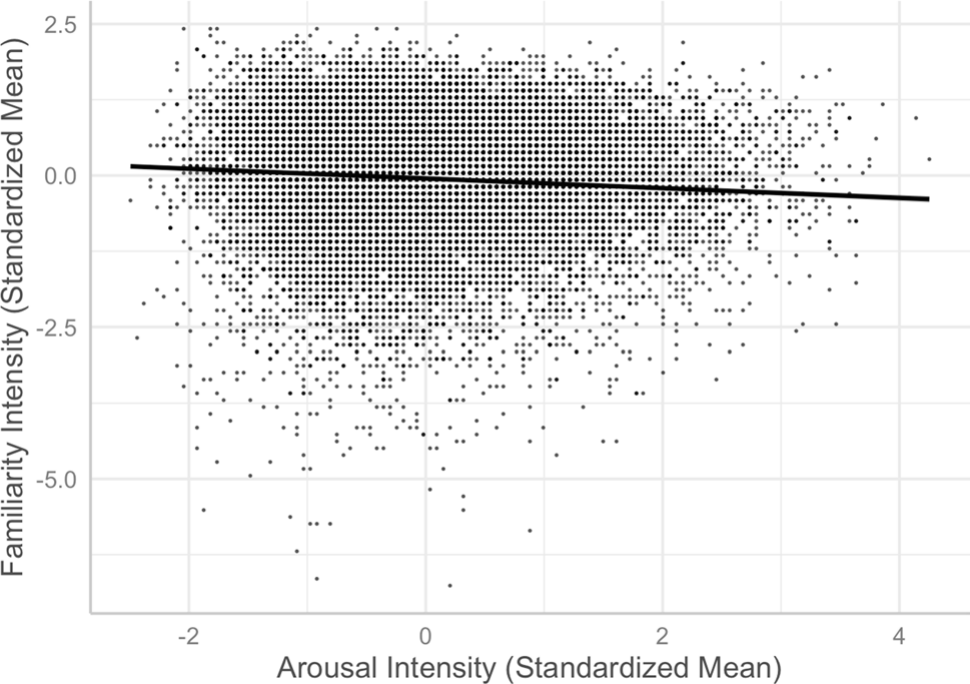
Arousal–familiarity relationship (Model 4)

We obtained a negative arousal–familiarity linear relationship (see Models 3 and 4), suggesting that more arousing words were rated less familiar (see Fig. 7). This was consistent with the findings in English (e.g., Warriner et al., 2013, but see Citron et al., 2014), but not the absence of such a relationship in Yee (2017).

#### **Arousal–concreteness**

**Fig. 8:**
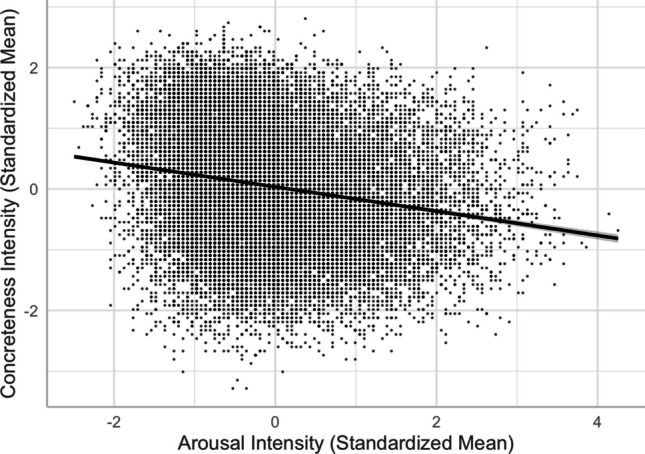
Arousal–concreteness relationship (Model 6)

Consistent with Xu et al. (2022), Yao et al. (2017), and Lv et al. (2023), we obtained a negative arousal–concreteness linear relationship (see Models 5 and 6), suggesting that abstract words were more arousing than concrete words (see Fig. 8). A similar finding was reported in English (e.g., Warriner et al., 2013), Indonesian (e.g., Sianipar et al., 2016), Polish (e.g., Imbir, 2016), and Spanish (e.g., Ferré et al., 2012; Guasch et al., 2016) (but see Montefinese et al., 2014, in which very abstract and concrete Italian words were rated calmer than those with a medium level of concreteness). This supports Vigliocco et al. (2009, 2014), that abstract words are associated with affective experience.

#### **Arousal–imageability**

**Fig. 9:**
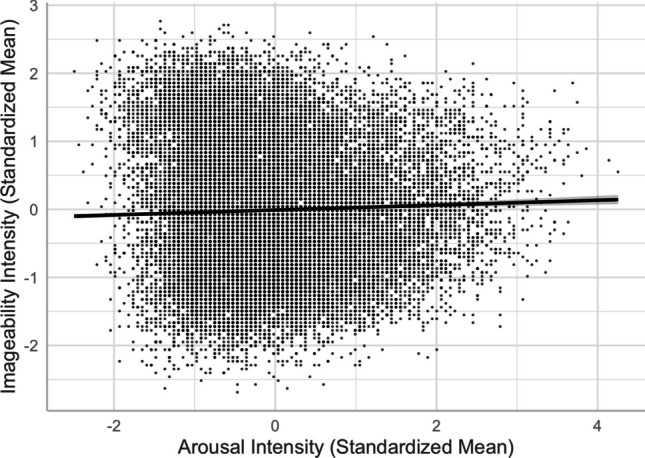
Arousal–imageability relationship (Model 8)

We obtained a positive arousal–imageability linear relationship (see Models 7 and 8). As depicted in Fig. 9, the more arousing words were rated as forming mental images more easily, which could be attributed to the intense experience associated with more arousing words that make it easier for individuals to form mental images. This was consistent with results obtained in English (e.g., Citron et al., 2014), but contrary to the weakly negative relationship in Yao et al. (2017; see also Guasch et al., 2016, for Spanish and the curvilinear relationship in Montefinese et al., 2014).

#### **Interrelationships among lexico-semantic variables**

**Fig. 10:**
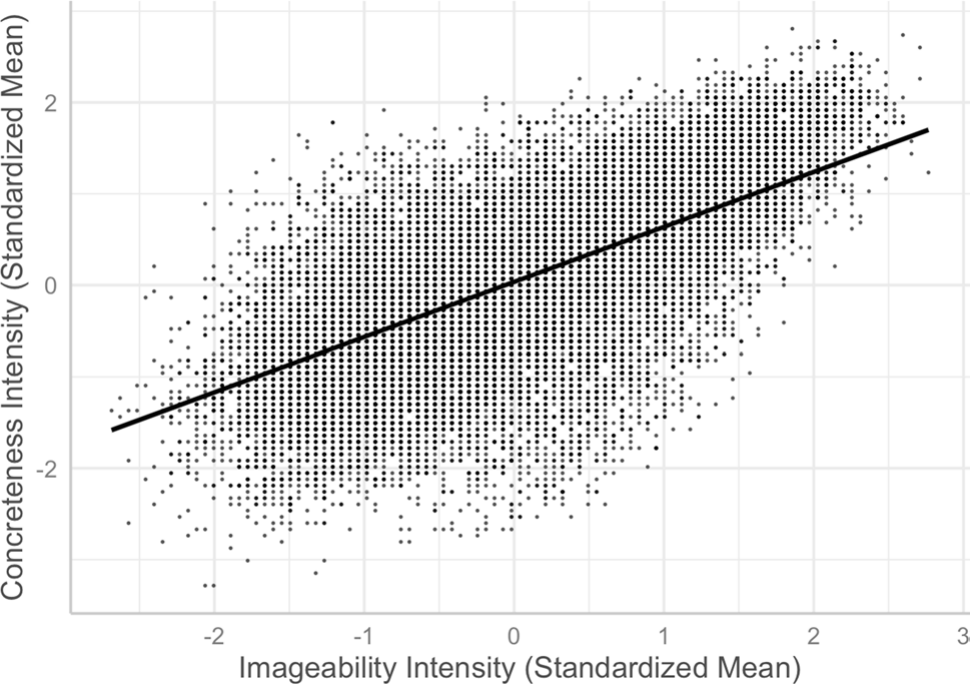
Imageability–concreteness relationship (Model 6)

**Fig. 11:**
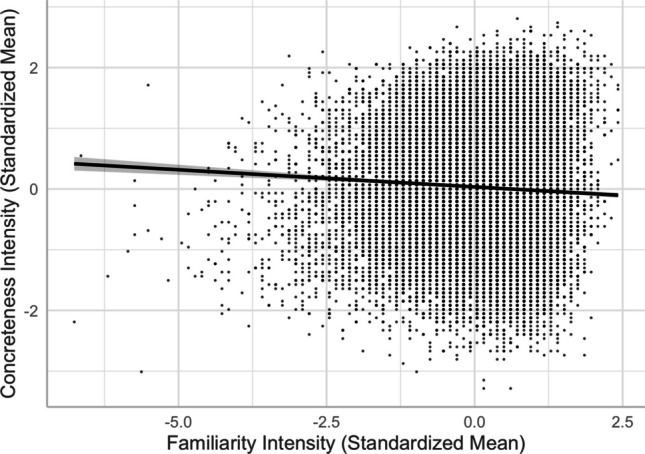
Familiarity–concreteness relationship (Model 6)

**Fig. 12:**
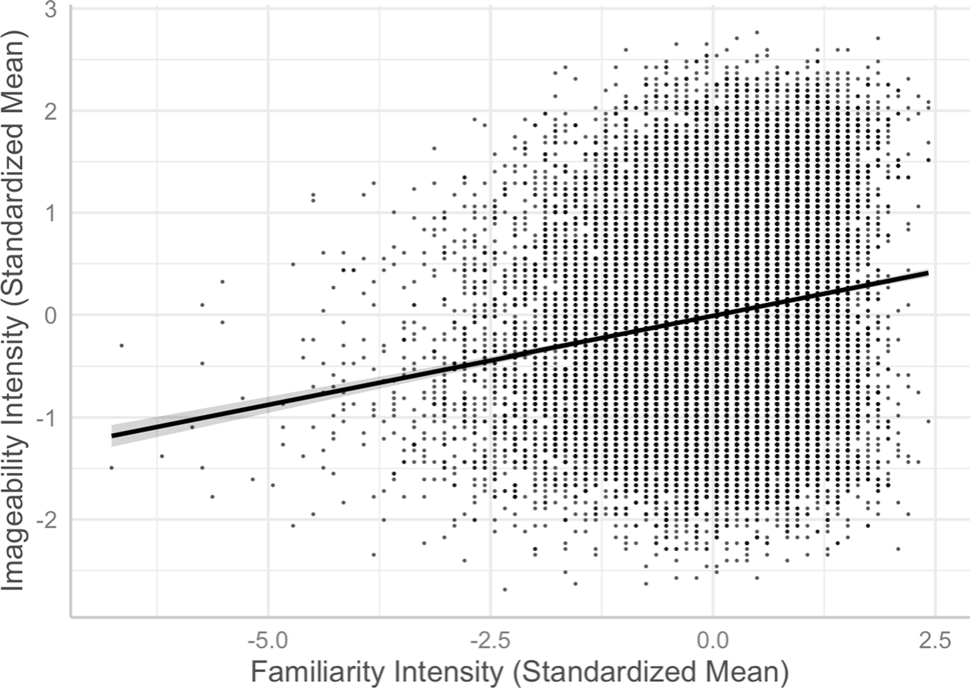
Familiarity–imageability relationship (Model 8)

We found a positive relationship between imageability and concreteness, a negative relationship between familiarity and concreteness, and a positive relationship between familiarity and imageability (see, e.g., Models 6 and 8 in Table 2). The imageability–concreteness relationship was in line with previous studies (e.g., Yee, 2017) and aligned with Paivio’s (1991) dual-coding theory, suggesting that concrete words were easier to imagine than abstract words (see Fig. 10). Similar to previous studies (e.g., Yao et al., 2017), the imageability–concreteness relationship was stronger than the familiarity–concreteness relationship and familiarity–imageability relationship. However, the weakly negative familiarity–concreteness relationship showed that concrete words were slightly *less* familiar than abstract words (see Fig. 11), inconsistent with the positive relationship reported by Yao et al. and Yee. On the other hand, these studies did not control for any other lexical variables as we did. In fact, we did find a slightly positive Pearson correlation (+.10) between familiarity and concreteness. This highlights the importance of controlling for extraneous variables in the analyses. Contrary to the negative familiarity–concreteness relationship, the familiarity–imageability relationship was positive, indicating that highly imageable words are *more* familiar than difficult-to-image words (see Fig. 12).

#### **Valence ambiguity**

Very few studies (e.g., Brainerd, 2018) have taken into account valence ambiguity, as quantified as the standard deviation of valence ratings across different raters. We included that as one of the controlling variables in our analyses and also examined how it could be associated with emotion and lexico-semantic variables.

#### **Valence and valence ambiguity**

**Fig. 13:**
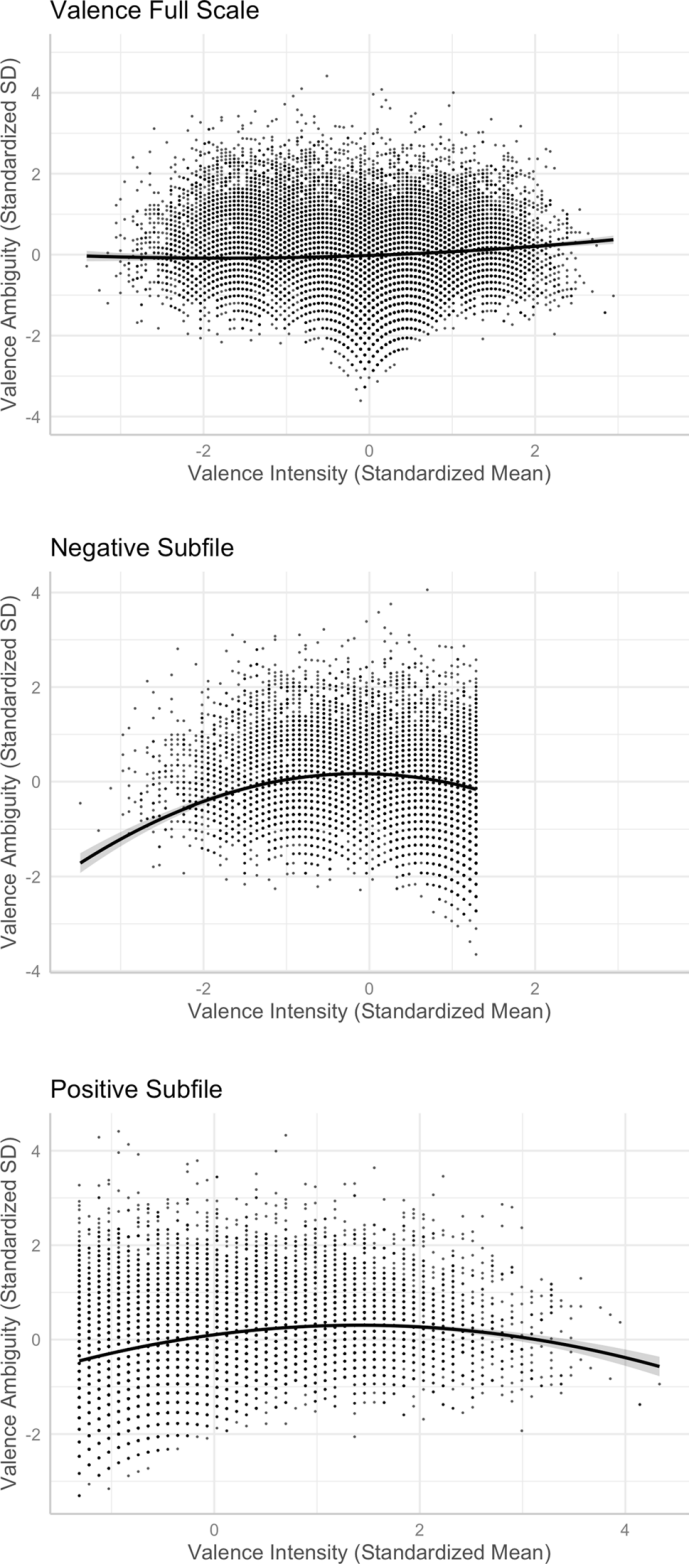
Valence–valence ambiguity relationship (Model 10)

As shown in Model 9, there was a positive linear relationship between valence and valence ambiguity [*R*^2^ = .1175, *F*(12,20205) = 225.2, *p* < .001], suggesting that positive words show larger valence ambiguity than negative words. To test the replicability of Brainerd et al.’s (2021a, 2021b) quadratic valence–valence ambiguity relationship, we added the squared valence term in the model and found that this explained more variance in valence ambiguity [Model 10, *R*^2^ = .1179, *F*(13,20204) = 208.9, *p* < .001, *ΔR*^2^ = .0005, *ΔF* = 11.925, *p* < .001]: negative and positive words were more ambiguous in valence than neutral words (see Fig. 13A), inconsistent with Brainerd et al.’s findings. While the Pearson correlation was indeed slightly negative (−.10) between valence and valence ambiguity, in Model 9 the linear relationship was positive when other variables were controlled. Thus, the discrepancy between Brainerd et al.’s findings and the current findings may be attributed to whether other lexical variables were controlled in the analyses.

On the other hand, the discrepancy might be due to how valence was conceptualized. While Brainerd et al. (2021a, 2021b) treated that as a bimodal variable, we treated valence as a unimodal variable. To test whether we could replicate Brainerd et al.’s findings by treating valence as a bimodal variable, we conducted additional analyses by splitting the valence scale into negative (mean < 5, *N* = 7390) and positive (mean ≥5, *N* = 12,288) subfiles, and examined the valence–valence ambiguity relationship separately for negative and positive subfiles.

In the negative subfile model, *R*^2^ = .1529, *F*(13,7916) = 111.1, *p* < .001, both the linear term (*β* = −.041, *SE* = .015, *p* < .01) and squared term (*β* = −.167, *SE* = .010, *p* < .001) of valence significantly predicted valence ambiguity. This indicates a concave downward relationship wherein valence ambiguity was higher for less negative words. However, as the words were rated as neutral (i.e., the right end of the *x*-axis in Fig. 13B), the valence–valence ambiguity relationship weakened. This was aligned with Brainerd et al.’s findings. In the positive subfile model, *R*^2^ = .1233, *F*(13,12274) = 133.9, *p* < .001, both the linear term (*β* = .288, *SE* = .011, *p* < .001) and squared term (*β* = −.102, *SE* = .007, *p* < .001) of valence significantly predicted valence ambiguity. This shows an inverted U-shaped relationship between valence ambiguity and positive valence. As depicted in Fig. 13C, valence ambiguity was highest at the middle range of the positive valence, again consistent with Brainerd et al.’s findings.

#### **Arousal and valence ambiguity**

**Fig. 14:**
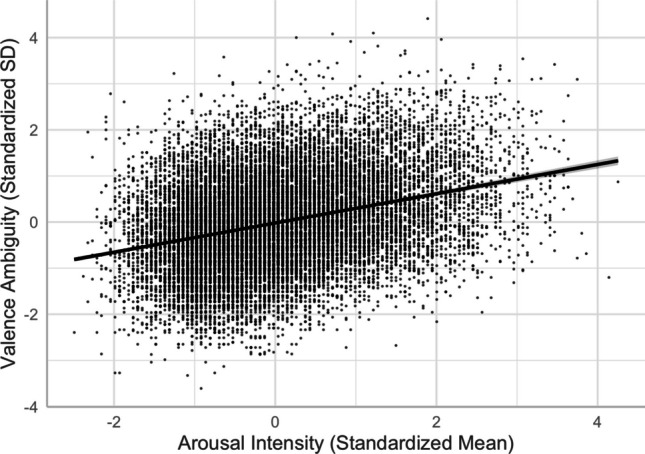
Arousal–valence ambiguity relationship (Model 10)

There was a positive linear arousal–valence ambiguity relationship (see Models 9 and 10), indicating that more arousing words were rated with more varied valence (i.e., high in valence ambiguity) than not-as-arousing words (see Fig. 14).

#### **Familiarity and valence ambiguity**

**Fig. 15:**
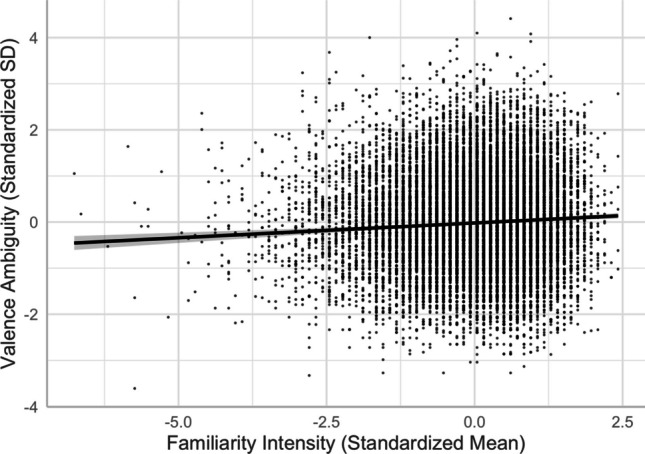
Familiarity–valence ambiguity relationship (Model 10)

There was a positive linear relationship between familiarity and valence ambiguity (see Models 9 and 10), indicating that more familiar words were rated with more varied valence (i.e., high in valence ambiguity) than unfamiliar words (see Fig. 15). This was aligned with Brainerd et al.’s (2021a) findings. They attributed the higher recall of valence-ambiguous (vs. unambiguous) words to their higher familiarity.

#### **Concreteness, imageability, and valence ambiguity**

**Fig. 16:**
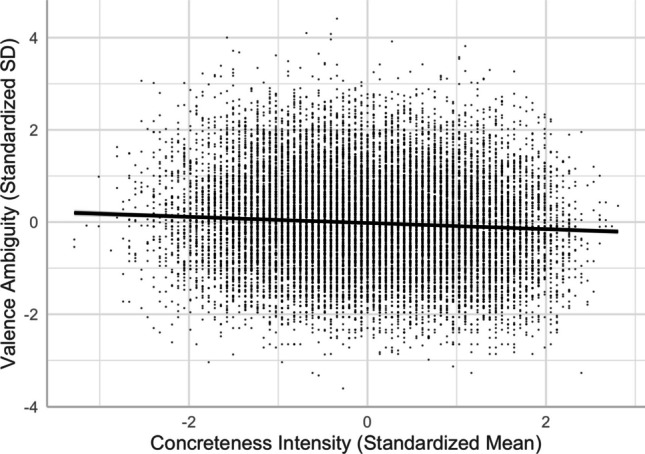
Concreteness–valence ambiguity relationship (Model 10)

**Fig. 17:**
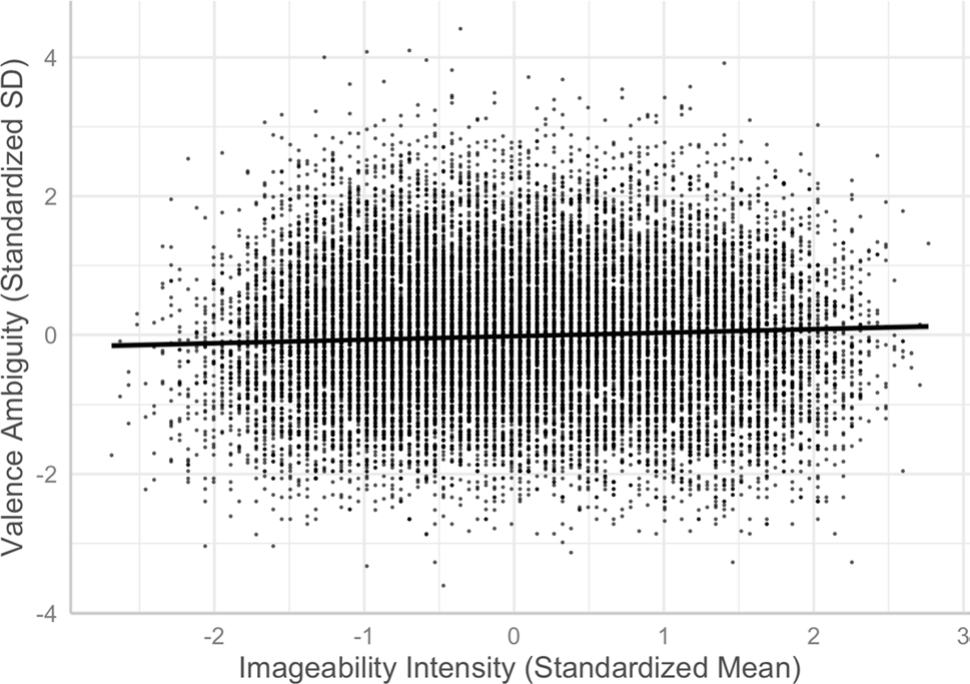
Imageability–valence ambiguity relationship (Model 10)

Similar to when familiarity or arousal was an outcome variable in regression models, concreteness and imageability predicted valence ambiguity differently. While the concreteness–valence ambiguity relationship was negative, the imageability–valence ambiguity relationship was positive. Abstract words and more imageable words showed higher valence ambiguity than concrete words and less imageable words, respectively (see Figs. 16 and 17).

### Emotional-ambiguity hypothesis

To test whether valence ambiguity and arousal ambiguity could moderate the valence–arousal relationship, as reported by Brainerd (2018, 2021b), we added the valence ambiguity × valence and valence ambiguity × valence^2^ interaction terms or arousal ambiguity × valence and arousal ambiguity × valence^2^ interaction terms in Model 2, which became Models 2a and 2b, respectively. Following Brainerd et al. (2021a, 2021b) where valence was treated as a bimodal variable, we ran additional analyses by splitting the valence scale into negative (mean ratings < 5, *N* = 7,390) and positive (mean ratings ≥ 5, *N* = 12,288) subfiles and examined the emotional-ambiguity hypothesis. Table 3 presents the results of these models.

#### **Valence ambiguity and valence–arousal relationship**

**Fig. 18:**
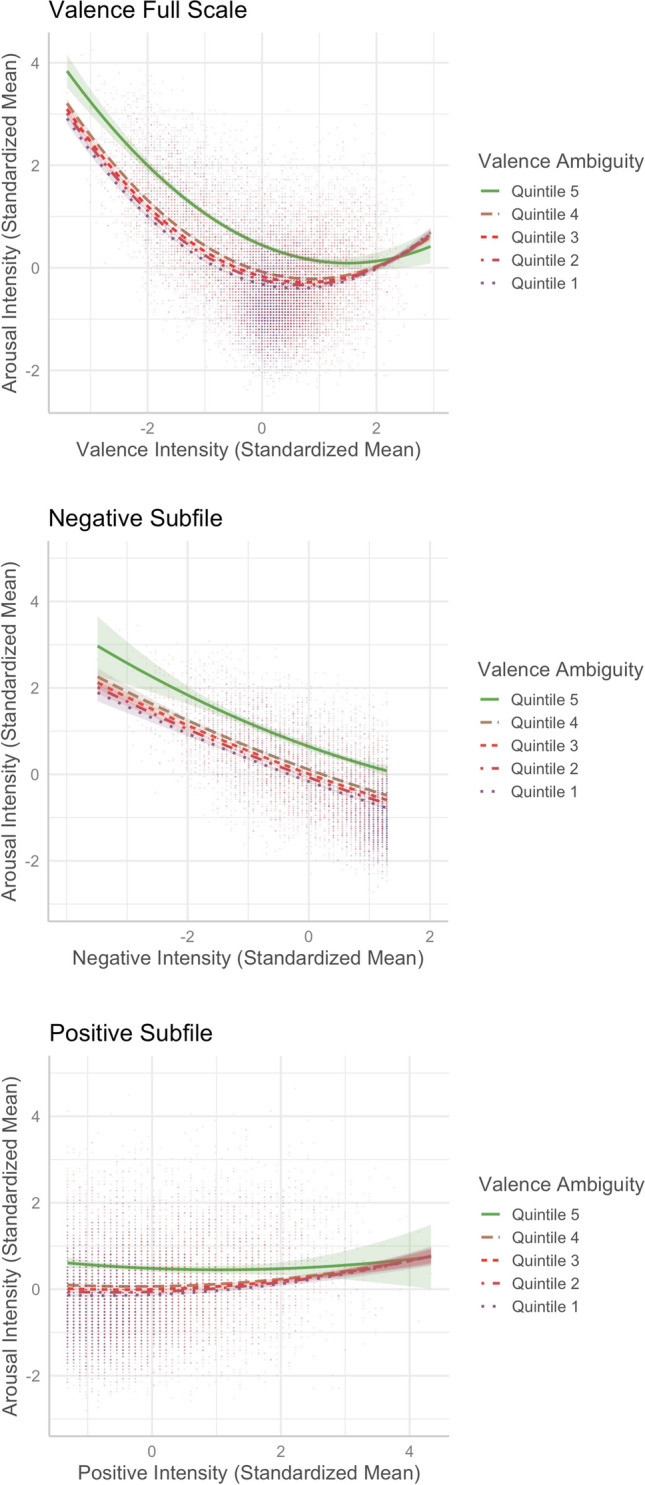
Moderation of valence ambiguity in the valence–arousal relationship (Model 2a)

Model 2a, which includes the valence ambiguity × valence and valence ambiguity × valence^2^ interaction terms, accounted for 0.11% more variance in arousal, *R*^2^ = .6108, *F*(15,20202) = 2116, *p* < .001, *ΔF* = 29.396, *p* < .001. When the valence scale was considered in full, we observed that valence ambiguity was less likely to impact the valence–arousal relationship for negative and neutral words, but for more positive words, the valence–arousal relationship was weaker when valence ambiguity was very high (i.e., in the fifth quintile, see Fig. 18A). The analyses by subfiles also showed that valence ambiguity moderated the valence–arousal relationship in the positive subfile model (*R*^2^ = .4981, *F*(15,12272) = 813.8, *p* < .001; Fig. 18C), but not in the negative subfile model (*R*^2^ = .5591, *F*(15,7914) = 671.3, *p* < .001; Fig. 18B). Only the valence ambiguity × valence interaction term significantly predicted arousal in the positive subfile (see Model 2a in Table 3).

#### **Arousal ambiguity and valence–arousal relationship**

**Fig. 19:**
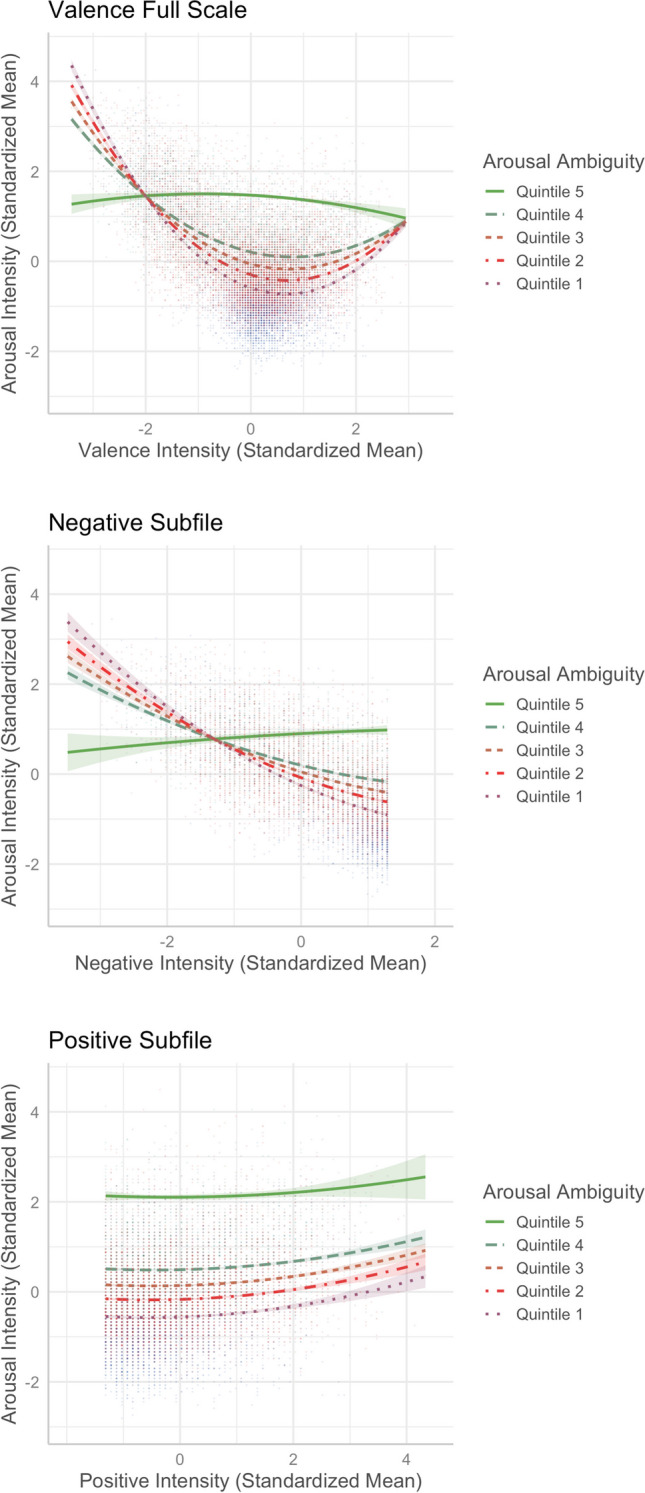
Moderation of arousal ambiguity in the valence–arousal relationship (Model 2b)

Model 2b, which includes the arousal ambiguity × valence and arousal ambiguity × valence^2^ interaction terms, explained 2.03% more variance in arousal, *R*^2^ = .63, *F*(15,20202) = 2296, *p* < .001, *ΔF* = 553.94, *p* < .001. When the valence scale was considered in full, we found that the asymmetric U-shaped valence*–*arousal relationship was changed, in the fifth quintile of arousal ambiguity (see Fig. 19A), showing that the valence*–*arousal relationship was different when arousal ambiguity was extremely high. When arousal ambiguity became lower, the valence*–*arousal relationship tended to be stronger (steeper) at both the negative and positive sides. The analyses by subfiles also showed that the valence*–*arousal relationship was moderated by arousal ambiguity in the negative subfile model, *R*^2^ = .5934, *F*(15,7914) = 772.6, *p* < .001 (see Fig. 19B), but not in the positive subfile model, *R*^2^ = .4978, *F*(15,12272) = 812.8, *p* < .001 (see Fig. 19C). Both the arousal ambiguity × valence and arousal ambiguity × valence^2^ interaction terms significantly predicted arousal in the negative subfile (see Model 2b in Table 3).

## Intensity–ambiguity relationship

Following Brainerd et al. (2021a, 2021b), we investigated the quadratic intensity–ambiguity relationship for normed arousal, familiarity, concreteness, and imageability of two-character Chinese words. Table 4 presents the model summaries for the regression analyses on the intensity–ambiguity relationships, while controlling for other variables (Models 11–18). For each ambiguity as an outcome variable, we first examined the linear relationship and then added the squared terms of the predictor variables to test the quadratic relationships.

### **Arousal and arousal ambiguity**

**Fig. 20:**
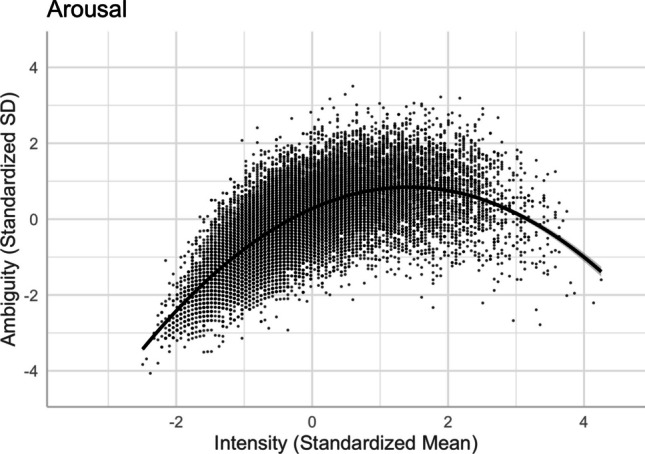
Arousal–arousal ambiguity relationship (Model 12)

There was a quadratic relationship between arousal and its ambiguity, as depicted in Model 12 [*R*^2^ = .4885, *F*(13,20204) = 1486, *p* < .001], consistent with Brainerd et al.’s (2021a, 2021b) findings, which suggested that the intensity–ambiguity relationship follows the quadratic function and can be explained by the categorical/quantitative model. As depicted in Fig. 20, arousal ambiguity was the highest when arousal intensity was at the mid-range. Relative to the linear model [Model 11, *R*^2^ = .3633, *F*(12,20205) = 962.1, *p* < .001], Model 12, with the squared term of arousal, significantly improved the model fit and accounted for more variance in arousal ambiguity (*ΔR*^2^ = .125, *ΔF* = 4949.2, *p* < .001).

### **Familiarity and familiarity ambiguity**

**Fig. 21:**
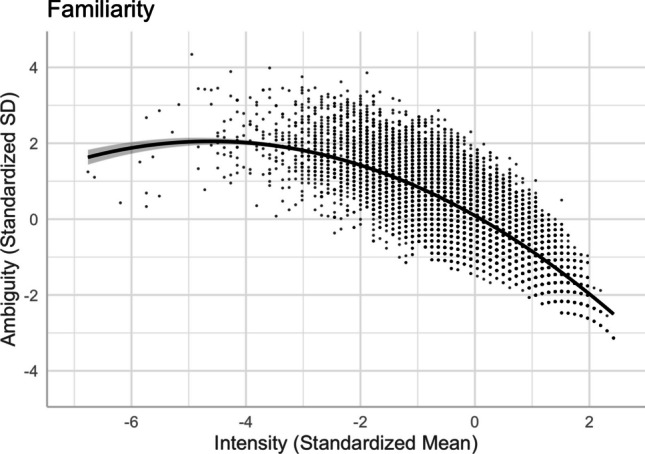
Familiarity–familiarity ambiguity relationship (Model 14)

There was a quadratic relationship between familiarity and its ambiguity, as depicted in Model 14 [*R*^2^ = .6578, *F*(13,20204) = 2990, *p* < .001]. Relative to the linear model [Model 13, *R*^2^ = .6342, *F*(12,20205) = 2922, *p* < .001], Model 14, with the squared term of familiarity, significantly improved the model fit and accounted for more variance in familiarity ambiguity (*ΔR*^2^ = .024, *ΔF* = 1394, *p* < .001). Figure 21 shows that the pattern was not fully aligned with the quadratic function, as less familiar words did not show lower familiarity ambiguity.

### **Concreteness and concreteness ambiguity**

**Fig. 22:**
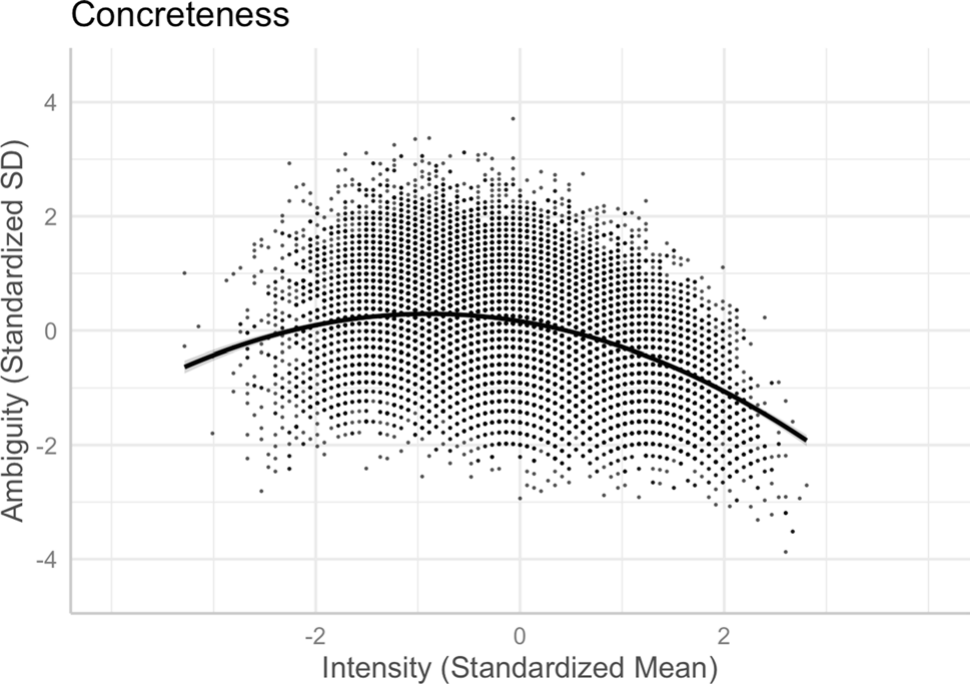
Concreteness–concreteness ambiguity relationship (Model 16)

There was a quadratic relationship between concreteness and its ambiguity [Model 16, *R*^2^ = .2135, *F*(13,20204) = 423.2, *p* < .001]. Relative to the linear model [Model 15, *R*^2^ = .1802, *F*(12,20205) = 371.2, *p* < .001], Model 16, with the squared term of concreteness, significantly improved the model fit and accounted for more variance in concreteness ambiguity (*ΔR*^2^ = .033, *ΔF* = 858.56, *p* < .001). The intensity–ambiguity relationship follows the quadratic function and can be explained by the categorical/quantitative model. As depicted in Fig. 22, concreteness ambiguity was higher for words with mid-range concreteness than for those with higher or lower concreteness.

### **Imageability and imageability ambiguity**

**Fig. 23:**
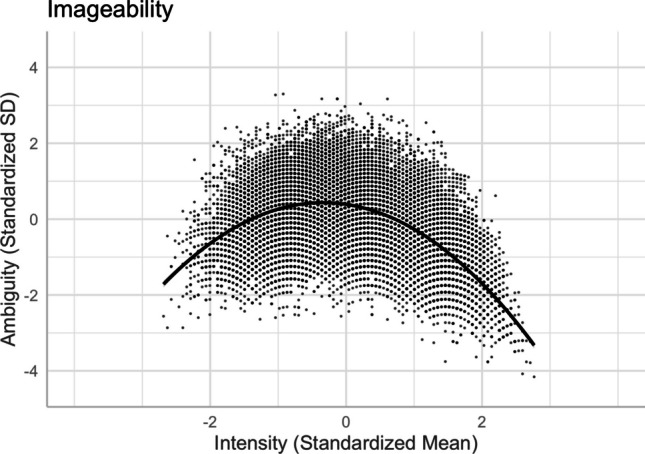
Imageability–imageability ambiguity relationship (Model 18)

There was a quadratic relationship between imageability and its ambiguity [Model 18, *R*^2^ = .273, *F*(13,20204) = 584.8, *p* < .001]. Relative to the linear model [Model 17, *R*^2^ = .098, *F*(12,20205) = 184.1, *p* < .001], Model 18, with the squared term of imageability, significantly improved the model fit and accounted for more variance in imageability ambiguity (*ΔR*^2^ = .175, *ΔF* = 4862.8, *p* < .001). The intensity–ambiguity relationship follows the quadratic function and can be explained by the categorical/quantitative model. As depicted in Fig. 23, imageability ambiguity was higher for words with mid-range imageability than for those with higher or lower imageability.

## Discussion

This norming study aimed to extend the psycholinguistic norms for Tse et al.’s. (2017, 2023) Chinese Lexicon Project. By collecting data on emotion variables (valence and arousal) and lexico-semantic variables (familiarity, concreteness, and imageability), we provide a reliable and valuable resource for future research in the field. Using a large pool of two-character Chinese words and controlling for other lexical variables (see Table 1), we examined the relationships among emotion variables and lexico-semantic variables, including their ambiguity measures (i.e., the standard deviation of the ratings for a lexical variable), which sheds light on the affective and semantic dimensions of two-character Chinese words (e.g., Brainerd’s, 2018, emotional-ambiguity hypothesis). In the following, we summarize and discuss the key findings of the current study.

First, we found an asymmetric U-shaped relationship between valence and arousal (see Fig. 3), indicating that negative words elicit stronger arousal, as they are associated with potential danger, while positive words are associated with feelings of safety and thus elicit weaker arousal than negative words (e.g., Citron et al., 2014). In the neutral–positive range (positive subfile), high valence ambiguity weakened the valence–arousal relationship (see Fig. 18B), while in the negative–neutral range (negative subfile), high arousal ambiguity weakened that relationship (see Fig. 19B). These partially support Brainerd et al.’s (2018) emotional-ambiguity hypothesis that the valence–arousal relationship decreases as valence ambiguity and arousal ambiguity increase.

Second, we revealed a U-shaped relationship between valence and familiarity (see Fig. 4), indicating that not only are positive words more familiar than neutral words, as attributed to the mere exposure effect (e.g., Zajonc, 2001), but negative words are also more familiar than neutral words, which can be explained by their being more attention-grabbing and memorable (e.g., Baumeister et al., 2001; Bowen et al., 2018).

Third, we demonstrated that not all emotion words were more abstract than neutral words. Positive words were perceived as slightly more abstract than negative words (see Fig. 5). While positive words might involve concepts or ideas that are less tangible or physically grounded (e.g., 涵養 “self-restraint” and 美妙 “amazing”), negative words might be linked to specific events, objects, or situations that evoke stronger sensory or perceptual representations (e.g., 非禮 “indecent assault” and 癌症 “cancer”). This might not be fully consistent with Vigliocco et al.’s (2014) view about the semantic representation of abstract words, which does not distinguish the role of positive versus negative emotion in abstract words.

Fourth, despite the typical moderate-to-strong association between concreteness and imageability (see Fig. 10), we observed an interesting contrast in their corresponding associations with valence, arousal, valence ambiguity, and familiarity. This provides evidence for the distinct constructs of these two seemingly highly correlated lexical variables—the differences in the concreteness-associated and imageability-associated relationship; that is, being negative and positive with arousal/valence/familiarity, respectively (see Figs. 5, 6, 8, 9, 11, and 12). For example, positive and more arousing words tend to be more abstract yet more imageable than negative and not-as-arousing words, respectively. This contrast clearly showed that concreteness and imageability are distinct constructs, echoing the findings of previous studies (e.g., Kousta et al., 2011) that concreteness and imageability should not be treated as interchangeable variables when investigating emotion word processing.

Fifth, we explored the role of valence ambiguity, which reflects the standard deviation of valence ratings, in the relationships among these lexical variables. For words with higher valence ambiguity, the high standard deviation of their valence rating suggests that they are likely associated with more varied concepts (e.g., both positive and negative valence) in semantic networks across individuals. For example, police station may be connected to department building, public safety, and crime—that is, concepts with neutral, positive, and negative valence, respectively. Additionally, this word may generate mixed emotions within an individual. These may also explain the differences in the direction of the valence ambiguity relationship associated with familiarity, concreteness, and imageability. As valence ambiguity allows for a broader range of possible meanings and conceptual links to be activated, valence-ambiguous words are perceived as more familiar and more likely to evoke abstract concepts that are not tied to specific sensory experience or concrete objects. Nevertheless, multiple possible interpretations and associations of valence-ambiguous words might also provide a rich context that facilitates the generation of mental images. It is noteworthy that the pattern of valence–valence ambiguity relationship was different when valence was treated as bimodal versus separated into negative and positive subfiles (see Fig. 13B and C). This followed the quadratic intensity–ambiguity relationship as proposed by Brainerd et al. (2021a, 2021b) that valence ambiguity is lower for more negative and more positive words. This finding can be explained by the categorical/quantitative model wherein participants tend to make categorical judgments for words with extremely strong valence but more fine-grained quantitative judgments for words in the middle range of valence.

Finally, we investigated the quadratic intensity–ambiguity relationship in other emotion and lexico-semantic variables. Replicating Brainerd et al.’s (2021a, 2021b) for our normed variables, we found that the ambiguity of arousal, concreteness, and imageability was smaller when the intensity (i.e., mean rating) was extremely low or extremely high (see Figs. 20, 22, and 23), similar to the valence ambiguity. However, for familiarity, although the intensity–ambiguity relationship was quadratic, similar to other lexical variables, familiarity ambiguity was not smaller when familiarity intensity was lower (see Fig. 21). This may be attributed to the fact that the two-character Chinese words included in Tse et al.’s (2017) Chinese Lexicon Project, despite varied familiarity, were quite well known to our participants, such that the word pool might not include those words that are highly unfamiliar to participants. The finding of familiarity–familiarity ambiguity might be better explained by the quantitative model than by the categorical/quantitative model (Brainerd et al., 2021a); that is, familiarity ambiguity tends to be negatively (and monotonically) correlated with familiarity intensity, rather than being higher when the familiarity intensity is extremely high or extremely low.

Before concluding the current paper, it is important to highlight some other lexical variables that were not normed in the current study, such as subjective age of acquisition (e.g., Xu et al., 2022), dominance (e.g., Warriner et al., 2013), and context availability (e.g., Altarriba et al., 1999). We exclude the subjective age-of-acquisition rating because participants might struggle to recall the specific age at which they acquired the words, thereby substantially lengthening the rating process given our large word pool (>25,000). We did not include dominance, which refers to the feeling of being in control or dominated (Bradley & Lang, 1999), since (i) it was not considered as a core dimension of emotion, as in the case of valence and arousal (e.g., Russell, 2003), and (ii) it was highly correlated with valence (e.g., Imbir, 2016; Moors et al., 2013). Context availability, defined as the ease with which a word can be associated with a specific context when it is used, was excluded because it was highly correlated with concreteness and imageability, but not associated with valence or arousal (e.g., Guasch et al., 2016; Yao et al., 2017). Despite the fact that the influence of these variables was not in line with the scope of our current research, we have to acknowledge that the ratings of these variables per se are important for future researchers, so they should be normed in future research.

## Conclusion

In the current study, we established normed ratings of typical emotion and lexico-semantic variables (valence, arousal, concreteness, imageability, and familiarity) of over 25,000 two-character Chinese words and demonstrated the interrelationships among emotion and lexico-semantic variables while controlling for other lexical variables (see Tables 2, 3 and 4 for summaries). The findings revealed several significant patterns, such as the asymmetric U-shaped valence–arousal relationship, where extremely negative words were rated as more arousing than extremely positive words. This curvilinear relationship could be moderated by valence and arousal ambiguities, generally consistent with Brainerd et al. (2021a, 2021b). We also replicated Brainerd et al.’s findings of quadratic relationships between normed variables (valence, arousal, concreteness, and imageability, except familiarity) and their ambiguities. Concreteness and imageability, despite being strongly correlated, demonstrated different relationships with arousal, valence, familiarity, and valence ambiguity, which to our knowledge has not been reported in the literature. Our study also underscores the importance of controlling for other variables when examining lexical relationships. For example, the change in the direction of the concreteness–familiarity relationship, from positive in pairwise correlation to negative in the regression model, highlights the importance of incorporating control variables to obtain a more accurate understanding of these relationships.

The current normed data with a large word pool (>25,000 Chinese words) will help future researchers gathering a wider range of emotion words while matching extraneous variables for their factorial-designed experiments. The normed ratings of emotion and lexico-semantic variables could also be used in the analyses of megastudy data. They could be included in item-level multiple regression analyses using a behavioral repository of lexical decision and speeded naming performance reported in Tse et al.’s (2017, 2023) Chinese Lexicon Project to examine the role of word valence, arousal, and valence ambiguity (while controlling for various orthographic, phonological, lexico-semantic variables) in visual word recognition. This should further our understanding of the roles of affective and semantic variables in visual word processing of two-character Chinese words.

Future research may consider comparing our Chinese norm with norms established in other languages in order to vary whether perception of word valence is necessarily universal. For example, Ho et al. (2015) compared their normed ratings in Chinese with English translation equivalents in ANEW. Based on a relatively small pool of words (< 1000), Ho et al. found that while some words showed consistent valence classifications in both English and Chinese, such as “confident” being classified as positive, there were cases where translations from English to Chinese did not maintain the same valence. For example, “crazy” was considered negative in English yet carried a positive connotation in its Chinese translation. Some words classified as neutral in English were sometimes perceived as positive or negative in Chinese, and vice versa. Other than showing that the perception of word valence might not necessarily be language-universal, these findings highlighted the importance of developing specific norms for Chinese words instead of relying on directly adopting words from English word norms and translating them for research purposes, which is also one of the goals in the current research.

## Preregistration

This study was not preregistered.

## Appendix

**Table 5:** Summary of affective norming studies

Study	Stimuli	Age	Number of words	Normed variables	Valence–arousal	Valence–familiarity	Valence–concreteness	Valence–imageability	Arousal–familiarity	Arousal–concreteness	Arousal–imageability
Chan & Tse, 2024 (Current Study)	Chinese words (two-character)	18–25 yo	20,218	Valence, arousal, familiarity, concreteness, imageability	Asymmetric U-shaped relationship Valence^2^ (β=.195) significantly added 5.6% of the variance to Model 1, including character and word frequency, the intensity and ambiguity variables of all other normed variables	Symmetric U-shaped relationship Valence^2^ (β=.050) significantly added 0.32% of the variance to Model 3, including character and word frequency, the intensity and ambiguity variables of all other normed variables	Concave downward relationship Valence^2^ (β= −.035) significantly added 0.16% of the variance to Model 5, including character and word frequency, the intensity and ambiguity variables of all other normed variables	Concave upward relationship Valence^2^ (β=.010) significantly added 0.02% of the variance to Model 7, including character and word frequency, the intensity and ambiguity variables of all other normed variables	β= −.039 (Obtained in Model 4, including character and word frequency, the intensity and ambiguity variables of all other normed variables, and valence^2^)	β = −.200 (Obtained in Model 6, including character and word frequency, the intensity and ambiguity variables of all other normed variables, and valence^2^)	β=.036 (Obtained in Model 8, including character and word frequency, the intensity and ambiguity variables of all other normed variables, and valence^2^)
Bradley & Lang, 1999	English adjectives, nouns, verbs	Not reported (undergraduate students)	1,034	Valence, arousal, and dominance	Symmetric U-shaped relationship (quadratic model not reported)	NA	NA	NA	NA	NA	NA
Citron et al., 2014	English adjectives, nouns, verbs	18–42 yo (mean=20.5, *SD*=3.98)	300	Valence, arousal, familiarity, imageability, and age of acquisition	Asymmetric U-shaped relationship Valence and valence^2^ significantly added 59% of variance to the model including familiarity, imageability, and age of acquisition	*r*=.29*** Valence (β=.13) significantly added 4% of variance to the model including arousal, frequency, length of phonemes and imageability	NA	*r*=.13* Valence (β=.11) significantly added 1% of variance to the model including arousal, familiarity, and age of acquisition.	r=.14* β=.16 (obtained in the model including imageability, age of acquisition, valence and valence^2^)	NA	*r*=.15** β=.09 (obtained in the model including familiarity, age of acquisition, valence, and valence^2^)
Eilola & Havelka, 2010	British English and Finnish nouns (includes taboo words)	English: 16–45 yo (mean=17.4, SD=2.71) Finnish: 16–35 (mean=18.7, *SD*=2.29)	210	Valence, emotional charge (arousal), familiarity, offensiveness, and concreteness	Symmetric U-shaped relationship for both languages The quadratic model explained 76% of the variance for English words, 71% of the variance for Finnish words	Not reported	Not reported	NA	Not reported	Not reported	NA
Ferré et al., 2012	Spanish nouns related to animals, people and objects	Mean=21.3 yo, *SD*=4.7	380	Valence, arousal, concreteness, and familiarity	Asymmetric U-shaped relationship (quadratic model not reported) Split: negative words (valence=1–4), *r*= −.65*** Positive words (valence=6–9), r=.20	Positive and neutral words were more familiar than negative words	No significant difference between negative, positive, and neutral words	NA	Not reported	High arousing words were more abstract than low arousing words	NA
Guasch et al., 2016	Spanish nouns, adjectives, interjections, and adverbs	Mean=21.54 yo, *SD*=4.56	1,400	Valence, arousal., concreteness, imageability, context availability, and familiarity	Asymmetric U-shaped relationship Split: negative words (valence=1–3.66), r= −.45*** Positive words (valence=6.34–9), r=.24***	*r*= −.02^a^	*r*= −.18*** ^a^	*r*= −.24*** ^a^	*r*= −.09***	*r*= −.14***	*r*= −.22***
Hinojosa et al., 2016	Spanish words	Mean=23.2 yo, *SD*=7.2	875	Valence, arousal, and concreteness	Asymmetric U-shaped relationship Valence^2^ significantly added 2.3% of variance to the linear model Split: negative words (valence < 5), *r*= −.37*** Positive words (valence > 5), *r*=.25***	NA	*r*= −.11***	NA	NA	*r*=.14***	NA
Ho et al., 2015	Chinese words (two-character)	12–17 yo	160	Valence, arousal, and threat	Symmetric U-shaped relationship he quadratic model explained 52.5% of variance	NA	NA	NA	NA	NA	NA
Imbir, 2015	Polish nouns, verbs, adjectives, adverbs and two-word phrases	18–49 yo (mean=20.74, *SD*=2.77)	1,586	Valence, arousal, dominance, origin, subjective significance, and source-of-experience	Symmetric U-shaped relationship (quadratic model not reported) Split: negative words (valence =< 5), *r*= −.40*** Positive words (valence > 5), *r*=.36***	NA	NA	NA	NA	NA	NA
Imbir, 2016	Polish nouns, verbs, adjectives, adverbs and two-word phrases	18–32 yo (mean=21.89, *SD*=1.91)	4,900	Valence, arousal, dominance, origin, subjective significance, concreteness^#^, imageability, and age of acquisition	Asymmetric U-shaped relationship. The quadratic model explained 48% of the variance	NA	*r*= −.078*** The quadratic model explained 32% of the variance (U-shaped relationship)	*r*=.126***	NA	*r*=.378*** ^#^	*r*= −.176*** The quadratic model explained 7.8% of the variance (inverted U-shaped relationship)
Lv et al., 2023	Chinese high-frequency words and phrases (did not restrict the number of character)	16–25 yo (mean=19.91, *SD*=1.21)	4,030	Valence, arousal, dominance, and concreteness	Asymmetric U-shaped relationship The quadratic model explained 21% of the variance Split: negative words (valence=1–3), *r*= −.225*** Positive words (valence=5–7), *r*=.268***	NA	Valence and Valence^2^ predicted 1.2% of the variance, *p*<.01 (quadratic, U-shaped relationship)	NA	NA	*r*= −.014*** Arousal (β= −.08, predicted 2% of the variance, *p*-value was not reported)	NA
Monnier & Syssau, 2014	French nouns and adjectives	18–40 yo (mean=20 years 9 month, *SD*= 2 years 4 months)	1,031	Valence and arousal	Symmetric U-shaped relationship The quadratic model explained 45% of variance Split: negative words (valence 1–4), *r*= −.55*** Positive words (valence 6–9), *r*=.50***	NA	NA	NA	NA	NA	NA
Montefinese et al., 2014	Italian nouns verbs, and adjectives	Valence, arousal, dominance: (mean =22.27 yo, *SD*=4.67) familiarity, imageability, and concreteness: (mean age=21.98, *SD*=4.6)	1,121	Valence, arousal, dominance, familiarity, imageability, and concreteness	Asymmetric U-shaped relationship The quadratic model explained 32.06% of the variance Split: negative words (valence < 5), β=−.479*** Positive words (valence > 5), β=.394***	NA	Not reported	Not reported	NA	concreteness and concreteness^2^ significantly added 2% of the variance to the model includes valence and dominance. (inverted U-shaped relationship)	imageability and imageability^2^ significantly added 1.4% of the variance to the model includes valence and dominance. (inverted U-shaped relationship)
Moors et al., 2013	Dutch nouns, adjectives, adverts, verbs	17–58 yo (mean=22.08, *SD*=4.49)	4,300	Valence, arousal, dominance, and age of acquisition	Symmetric U-shaped relationship (quadratic model not reported)	NA	NA	NA	NA	NA	NA
Quadflieg et al., 2014	French adjectives	18–27 yo (mean=20.2)	875	Valence, intensity (arousal), concreteness, familiarity, temporal stability, and visibility	Symmetric U-shaped relationship The quadratic model explained 34% of the variance	*r*=.30*** The quadratic model explained 27% of the variance	*r*=.03 The curvilinear relationship was nonsignificant	NA	*r*= −.05	*r*= −.11***	NA
Redondo et al., 2007	Spanish nouns, verbs, and adjectives	18–25 yo (mean=21.5, *SD*=1.81)	1,034	Valence, arousal, dominance, familiarity, concreteness, and imageability	Symmetric U-shaped relationship The quadratic model explained 27.1% of the variance	Not reported	Not reported	Not reported	Not reported	Not reported	Not reported
Riegel et al., 2015	Polish nouns, verbs, and adjectives	20–52 yo (mean=23.7, *SD*=4.9)	2,902	Valence, arousal, and imageability	Symmetric U-shaped relationship The quadratic model explained 48% of the variance	NA	NA	*r*=.21***	NA	NA	*r*=.03
Sianipar et al., 2016	Indonesian words	17–42 yo (mean=20)	1,490	Valence, arousal, dominance, predictability, subjective frequency, and concreteness^#^	Symmetric U-shaped relationship The quadratic model explained 34% of the variance. Split: negative words (valence=2.08–4.56), *r*= −.42*** Positive words (valence=5.7–7.92), *r*=.32***	NA	*r*=.31*** ^#^ The quadratic model explained 9.8% of the variance.	NA	NA	*r*=.36*** ^#^	NA
Soares et al., 2012	Portuguese nouns, adjectives, adverb, verb, and interjection	mean = 22.92 yo, *SD*=5.41	1,034	Valence, arousal, and dominance	Asymmetric U-shaped relationship The quadratic model explained 39% of the variance. Split: negative words (valence<5), *r*= −.58*** Positive words (valence>5), *r*=.27***	NA	NA	NA	NA	NA	NA
Söderholm et al., 2013	Finnish nouns	16–77 yo (mean=32.91, *SD*=14.5)	420	Valence and arousal	Asymmetric U-shaped relationship The quadratic model explained 56.2% of the variance. Split: negative words (valence=1–3), *r*=.719*** Positive words (valence=5–7), r=.10	NA	NA	NA	NA	NA	NA
Võ et al., 2009	German nouns, verbs, and adjectives	mean= 27.14 yo, *SD*=9.11	2,900	Valence, arousal, and imageability	Asymmetric U-shaped relationship The quadratic model explained 37% of the variance. Split: negative words (valence −3 to 0), β=−.64*** Positive words (valence 0 to +3), β=.12***	NA	NA	Not reported	NA	NA	Not reported
Wang et al., 2008	Chinese nouns (two-character)	18-21 yo	1,500	Valence, arousal, dominance, appulsion, and familiarity	Not reported (Correlation plot showed symmetric U-shape)	Not reported (Correlation plot showed positive relationship)	NA	NA	NA	NA	NA
Warriner et al., 2013	English nouns, verbs, and adjectives (high frequency words)	16–87 yo 11% < 20 yo 45% 21–30 yo 21% 31–40 yo 11% 41–49 yo 12% >50 yo	13,915	Valence, arousal, and dominance	Symmetric U-shaped relationship The quadratic model explained 14.3% of the variance. Split: negative words (valence < 4), *r*= −.293*** Positive words (valence > 6, *r*=.273***	*r*=.206***	*r*=.105***	*r*=.161***	*r*= −.193***	*r*= −.258***	*r*= −.012 (Figure showed words with extremely low or high imageability tend to be rated calmer, whereas words with moderate imageability levels tend to be rated more arousing.)
Xu et al., 2022	Chinese words (two-, three-, and four-character)	18–62 yo	11,310	Valence and arousal	Asymmetric U-shaped relationship (quadratic model not reported) Valence: *r*= −.10** Valence^2^: *r*=.52***	NA	*r*=.01^#^	NA	NA	*r*=.20** ^#^	NA
Yao et al., 2017	Chinese nouns, adjectives, and verbs (two-character)	18–21 yo	1,100	Valence, arousal, concreteness, imageability, familiarity, and context availability	Symmetric U-shaped relationship The quadratic model explained 39.8% of the variance.	Valence and Valence^2^ added 15.9% of the variance, *p*<.001 (quadratic, U-shape)	Valence and Valence^2^ added 2.2% of the variance, *p*<.001 (quadratic, inverted U-shaped)	Valence and Valence^2^ added 0.7% of the variance, *p*<.001 (quadratic, inverted U-shaped)	Not reported	Arousal predicted 1.5% of the variance, *p*<.01 (Negatively related, but regression coefficient was not reported.)	Linear model: nonsignificant (*p*=.47) Partial correlation: −.06 (*p*=.05)
Yee, 2017	Chinese low-/ medium-frequency nouns (two-character)	Not reported (undergraduate students)	292	Valence, arousal, concreteness, imageability, and familiarity,	Symmetric U-shaped relationship The quadratic model explained 46.3% of the variance.	*r*=.38***	*r*= −.12*	*r*= −.01	*r*= −.11	*r*= −.02	*r*=.02

yo = years old, r=Pearson correlation coefficient, β=regression coefficient

***p<.001, **p< .01, *p<.05, NA = Not available

# Concreteness rating ranged from concrete to abstract

@ Imbir (2016) mentioned the arousal–imageability relationship was quadratic, but they did not report the statistics

^a^ Guasch et al.’s (2016) valence scale was recoded as emotional loaded (positive and negative) vs. non-emotional loaded (neutral)

see Table 5

## Data Availability

Data and analysis code reported in the paper are available at: https://osf.io/hwkv7.

## Footnotes

1 Lv et al. (2023) did not specify the number of two-character words included in their normed lists.

2 We also analyzed the valence–arousal relationship using Yao et al.’s (2017) criteria for negative words (1-4, *N* = 3,286, valence mean = 3.38, *SD* = .413; arousal mean = 4.94, *SD* = .725) and positive words (6–9, *N* = 2,861, valence mean = 6.46, *SD* = .337; arousal mean = 3.79, *SD* = .706). We found that negative words were negatively correlated with arousal (−.433, *p* < .001), while positive words were positively correlated with arousal (+.173, *p* < .001), replicating the asymmetric U-shaped relationship wherein negative words were more arousing than positive words.
